# Diagnosis, Treatment and Long-Term Management of Vitamin B12 Deficiency in Adults: A Delphi Expert Consensus

**DOI:** 10.3390/jcm13082176

**Published:** 2024-04-10

**Authors:** Rima Obeid, Emmanuel Andrès, Richard Češka, Babak Hooshmand, Rosa-Maria Guéant-Rodriguez, Gabriel Ioan Prada, Jarosław Sławek, Latchezar Traykov, Binh Ta Van, Tamás Várkonyi, Karlheinz Reiners, The Vitamin B12 Consensus Panelists Group

**Affiliations:** 1 Department of Clinical Chemistry and Laboratory Medicine, Saarland University Hospital, 66421 Homburg, Germany; 2 Department of Internal Medicine, Diabetes and Metabolic Diseases, Hôpitaux Universitaires de Strasbourg, 67091 Strasbourg, France; 3 IIIrd Department of Internal Medicine, Center of Preventive Cardiology, University General Hospital, Charles University in Prague, 110 00 Prague, Czech Republic; 4 Aging Research Center, Karolinska Institute, 171 65 Stockholm, Sweden; 5 Department of Neurology, Benedictus Klinikum Tutzing, 82327 Tutzing, Germany; 6 Department of Psychiatry and Psychotherapy, Ludwig Maximillian University Hospital, 80539 Munich, Germany; 7 INSERM, UMR_S1256, NGERE–Nutrition, Genetics, and Environmental Risk Exposure, Faculty of Medicine of Nancy, University of Lorraine, 54500 Vandoeuvre-lès-Nancy, France; 8 Department of Biochemistry, Molecular Biology, Nutrition, and Metabolism, University Hospital of Nancy, 54000 Vandoeuvre-lès-Nancy, France; 9 Clinical Department of the National Institute of Gerontology and Geriatrics “Ana Aslan”, University of Medicine and Pharmacy “Carol Davila”, 011241 Bucharest, Romania; 10 Department of Neurology & Stroke, St. Adalbert Hospital, 80-462 Gdańsk, Poland; 11 Department of Neurological-Psychiatric Nursing, Faculty of Health Sciences, Medical University of Gdańsk, 80-210 Gdańsk, Poland; 12 Department of Neurology, University Hospital “Alexandrovska”, Medical University, 1431 Sofia, Bulgaria; 13 Vietnam Institute of Diabetes and Metabolic Disorders, Hanoi Medical University, Hanoi 116001, Vietnam; 14 Department of Internal Medicine, University of Szeged, 6720 Szeged, Hungary; 15 Consultant in Neurology and Clinical Neurophysiology, 41844 Wegberg, Germany

**Keywords:** diagnosis, neuropathy, cognitive decline, anemia, treatment, vitamin B12 deficiency

## Abstract

**Background/Objectives**: Vitamin B12 deficiency can cause variable symptoms, which may be irreversible if not diagnosed and treated in a timely manner. We aimed to develop a widely accepted expert consensus to guide the practice of diagnosing and treating B12 deficiency. **Methods**: We conducted a scoping review of the literature published in PubMed since January 2003. Data were used to design a two-round Delphi survey to study the level of consensus among 42 experts. **Results**: The panelists agreed on the need for educational and organizational changes in the current medical practices for diagnosing and treating B12 deficiency. Recognition of clinical symptoms should receive the highest priority in establishing the diagnosis. There is agreement that the serum B12 concentration is useful as a screening marker and methylmalonic acid or homocysteine can support the diagnosis. Patient lifestyle, disease history, and medications can provide clues to the cause of B12 deficiency. Regardless of the cause of the deficiency, initial treatment with parenteral B12 was regarded as the first choice for patients with acute and severe manifestations of B12 deficiency. The use of high-dose oral B12 at different frequencies may be considered for long-term treatment. Prophylactic B12 supplementation should be considered for specific high-risk groups. **Conclusions**: There is a consensus that clinical symptoms need to receive more attention in establishing the diagnosis of B12 deficiency. B12 laboratory markers can support the diagnosis. The severity of clinical symptoms, the causes of B12 deficiency, and the treatment goals govern decisions regarding the route and dose of B12 therapy.

## 1. Introduction

Vitamin B12 (cobalamin) deficiency can affect several organs, such as the bone marrow and the peripheral and central nervous systems [1]. The signs and symptoms of deficiency are variable and mostly nonspecific. Patients often seek help in primary medical care.

Dietary sources of vitamin B12 are foods of animal origin such as red meat, liver, fish, and dairy products. Insufficient dietary intake of vitamin B12 (<4–5 µg/day) can cause vitamin B12 deficiency. In addition, intestinal absorption of vitamin B12 requires the release of vitamins from food proteins, normal secretion and function of intrinsic factor, and appropriate gastrointestinal acidity. Malabsorption of vitamin B12 is the main cause of clinically manifested vitamin B12 deficiency among adults. Pernicious anemia (lack of intrinsic factor or antibodies against intrinsic factor), atrophic gastritis, and other gastroenteric diseases can cause B12 malabsorption. People with vitamin B12 deficiency caused by malabsorption disorders show gastrointestinal symptoms (episodes of abdominal distress, distension, nausea, and diarrhea) [2] in addition to symptoms such as neuropathy that is caused by the deficiency itself [3].

Manifestations related to the hematopoietic system such as megaloblastic anemia can be diagnosed using widely available laboratory markers. Approximately 30–50% of patients with vitamin B12 deficiency have some degree of neurological involvement [4]. Psychiatric symptoms such as mental and mood disorders and cognitive dysfunction are also common in people with B12 deficiency [4,5,6]. If the diagnosis of neuropsychological manifestations of vitamin B12 deficiency is delayed, symptoms may become irreversible [7,8,9] within variable time intervals (months to years), depending on residual B12 stores in the liver.

The serum concentration of vitamin B12 is a commonly used marker of vitamin B12 status. The majority of vitamin B12 in blood is bound to haptocorrin, which is not available for B12-dependent enzymatic reactions in cells. Holotranscobalamin is vitamin B12-bound to transcobalamin and constitutes a small fraction of total serum B12, which is biologically active. Adenosylcobalamin and methylcobalamin deficiency results in increased plasma concentrations of methylmalonic acid and homocysteine, respectively. Circulating vitamin B12 may not be lowered in all patients with B12 deficiency [10]. Using metabolic markers of B12 status like methylmalonic acid and homocysteine to aid in the diagnosis of clinically manifested vitamin B12 deficiency has advantages, but also shortcomings [10,11], such as the high costs of measurements, the limited availability, and the impact of renal insufficiency on the concentrations.

The severity of vitamin B12 deficiency symptoms at first presentation affects the choice of treatment modality. For example, initial parenteral treatment is used when the clinical symptoms are severe [12]. For the long-term management of pernicious anemia after B12 replenishment, parenteral treatment with B12 every 1–3 months seems sufficient to prevent a relapse [13,14,15]. More recent studies have suggested that the effectiveness of parenteral and high-dose oral B12 (1 mg/d) is similar in correcting serum concentrations of vitamin B12, though the evidence is of low quality. Nevertheless, high-dose oral B12 can likely be used for maintenance treatment in many patients [13,14,15].

The UK National Institute for Health and Care Excellence (NICE) released guidelines intended to facilitate the medical care of people with vitamin B12 deficiency specifically in the UK [16]. However, due to uncertainties and lack of evidence on multiple aspects related to the diagnosis and treatment of vitamin B12 deficiency, widely accepted guidelines are still needed due to between-country differences in medical practices, healthcare systems, and generally limited resources. The aim of this study was to establish an internationally acceptable consensus based on a review of the literature published over the past 20 years.

## 2. Materials and Methods

Following a scoping review of the literature, we summarized consistent results related to aspects of the diagnosis and treatment of vitamin B12 deficiency. In areas with insufficient evidence, we used a modified Delphi method to establish a practice-oriented expert consensus that can guide the medical care of people with vitamin B12 deficiency and those at risk for deficiency.

### 2.1. Methods of the Scoping Review

A literature search was conducted in PubMed on 10 February 2023 according to the search terms shown in Table S1 and by applying language (only English) and time filters (from 1 January 2003). The search was performed according to a detailed protocol (unpublished) that was prepared before starting the study. Observational and interventional studies, systematic reviews, and meta-analyses were qualified. Narrative reviews, animal studies, in vitro studies, case reports, case series including <3 cases, letters to the editor, and reports on inherited causes of vitamin B12 deficiency were excluded. The detailed inclusion and exclusion criteria are shown in Table S2. The initial search identified 432 potentially relevant articles (Figure S1). After screening the titles and abstracts, 170 articles qualified for full-text screening (Table S3). Thereafter, 19 articles were excluded for various reasons, and the remaining 151 articles were categorized by topics (Table S3). We aimed to address the following topics in the consensus: (1) clinical manifestations of B12 deficiency (neurological and hematological endpoints), (2) risk groups, (3) causes of B12 deficiency, (4) laboratory diagnosis of B12 deficiency, and (5) treatment and long-term management of the deficiency.

Key information was extracted from each publication (whenever relevant) on the study question, characteristics of the participants, form of intervention, biomarkers used to diagnose B12 deficiency, clinical tests, outcomes, and key findings. In addition, we defined key open questions raised by each article. The extracted data was used to (1) identify topics with sufficient between-study agreement and (2) for a stepwise prioritization of open questions to be used in a Delphi survey. The Delphi method was used to determine experts’ consensus on the open questions [17].

### 2.2. Recruitment of Scientific Board Members and Panelists

The scientific board members were invited by the leading authors (RO and KR) based on medical expertise, practical experience in the field of B12 deficiency, publication records, seniority, and willingness to participate. Nine out of twelve experts accepted the invitation to serve as scientific board members. The remaining three experts declined due to conflicts of interest. The scientific board consisted of one pharmacist and nine medical doctors (four from neurology/neuropsychiatry and five from internal medicine). The scientific board was responsible for planning the study, setting and refining the survey questions, and discussing and interpreting the results of the surveys. The board met once prior to the first survey round (June 2023) and once after the second survey round (November 2023). To ensure the neutrality of the results, the scientific board members were not eligible to participate in the surveys.

The panelists (the survey participants) were recruited through different channels. First, all corresponding authors of the 151 original articles identified through the literature search were invited to participate. Second, experts were identified through professional networks of board members, in addition to key opinion leaders in the field who had not published original studies in the last 20 years. Forty-six panelists agreed to participate in the survey. The first survey round was completed by 46 panelists, while the second survey was completed by 42 panelists (4 non-responders in the second round). Two online surveys were conducted using the SurveyMonkey Genius^®^ program. For both surveys, the responses of the panelists were anonymous.

### 2.3. Conduction of the Delphi Survey

A two-round web-based survey was conducted between June 2023 and October 2023. The survey included general questions related to the professional and geographical background of the participants, experience in the vitamin B12 field, and questions related only to the topics of vitamin B12 diagnosis and treatment/management (Table S4). The first survey consisted of questions offering single or multiple answers, including numerical estimates when appropriate. Several open questions were included where the panelists were able to provide their individual input as free text (Table S4).

The second survey aimed to test the consistency of the responses of the panelists on the topics that achieved consensus in the first survey (Table S5). In the second survey, the panelists rated each of the questions on a five-point Likert scale (strongly agree, agree, neutral, disagree, strongly disagree). We included the option “I do not have the expertise to answer this question” as a possible answer in both rounds of the survey. The data analysis included only the responses of people who considered themselves qualified to answer a specific question.

### 2.4. Data Analysis and Consensus Definition

After returning the answers of the first survey round, we calculated the mean and 80% confidence intervals (CIs) of the percentage of panelists who agreed with each of the answers. The first survey included widely formulated questions that needed to be prioritized for a more focused second survey. Therefore, wider uncertainty intervals (80% CIs) were used in the first survey cycle. Only answers with a level of agreement of at least 50% (as a lower bound of the 80% CI) were revised and used in the second round of the survey. In the second round of the survey, the upper two categories on a five-point Likert scale (the “agree” and “strongly agree” categories) were combined. A question was considered to reach a consensus if the combination of the “agree” and “strongly agree” categories constituted at least 50% of the total responses (50% as the lower bound of the 95% CI of the mean response). Questions that did not achieve consensus were considered topics with uncertainty or candidate topics for future research. The mean percentage and the 80% or 95% CIs of the panelists who agreed with the answers of the survey rounds are shown in Tables S4 and S5, respectively.

## 3. Results

### 3.1. Vitamin B12 Deficiency in the Medical Literature

Several topics showed consistent results between the studies (Table S6). Macrocytic anemia is not related to the presence or absence of neurological symptoms or their severity [11,18,19,20,21]. The serum concentration of vitamin B12 is commonly used as a primary marker of vitamin B12 status. However, 30–40% of people with neurological or hematological symptoms related to B12 deficiency may have normal vitamin B12 concentrations [22,23,24,25]. Serum vitamin B12 concentrations below 148 pmol/L (or 156 pmol/L in certain studies) are commonly considered to suggest a frank deficiency [26,27,28,29,30]. Serum B12 concentrations between 148 and 220 pmol/L or 260 pmol/L are often considered to indicate mild deficiency [31,32,33]. Some studies measured plasma homocysteine concentrations [7,34] or both homocysteine and methylmalonic acid [35,36,37] along with serum B12. For homocysteine and methylmalonic acid concentrations, there is a large variability in the cutoff values and combinations with other markers [11]. Low serum B12 concentrations and elevated methylmalonic acid [38] are not consistently correlated with the presence or severity of polyneuropathy or the clinical response to B12 treatment [39]. Vitamin B12 treatment increases serum B12 concentrations and decreases methylmalonic acid and homocysteine levels [18], but the normalization of these markers does not always correspond to clinical improvement. In general, determining the severity of B12 deficiency and the response to treatment primarily relies on evaluating clinical symptoms.

The use of the glucose-lowering drug metformin is causally related to lowered serum concentrations of vitamin B12 [40,41,42,43,44,45,46]. The effect has been shown as early as 3 months after starting the drug [47]. Low serum vitamin B12 concentrations in people using metformin are associated with hyperhomocysteinemia [48], a higher incidence of neuropathy [49,50], and worse neuropathy scores [51]. A recently published longitudinal study showed that people using metformin had a greater risk of neuropathy [HR = 1.84 (95% CI, 1.62, 2.10)] than those not using metformin [49]. There was a dose‒response association between the daily dose of metformin and a greater risk of neuropathy [49], which could suggest that low vitamin B12 concentrations are a direct cause of neuropathy in those patients. Vitamin B12 supplementation can increase serum B12 concentrations in users of metformin [47,52], but it remains unclear whether it may reduce neuropathy risk.

Vitamin B12 deficiency is common in elderly people [53,54] and is often explained by food-cobalamin malabsorption [55,56]. There is currently no clear evidence that vitamin B12 deficiency has an etiological role in frailty [54] or sarcopenia [57] in elderly people. The presence of some gastrointestinal disorders constitutes a major risk factor for vitamin B12 deficiency. Pernicious anemia often occurs parallel to other autoimmune disorders [27] and in people who are first-degree relatives of patients with this condition [58]. Positive antibodies against intrinsic factor or parietal cell antibodies are found in 30–50% of patients with neurological manifestations related to B12 deficiency [3,4]. Pernicious anemia may be associated with a deficiency of multiple nutrients, such as folate and iron [59]. Lowered serum vitamin B12 has been reported in people with atrophic gastritis [2,60], Crohn’s disease [61,62,63], celiac disease [64], infection with *H. pylori* [65], gastric surgeries [66,67,68,69,70], and chronic treatment with proton pump inhibitors [71]. Up to 70% of patients who undergo gastrectomy may develop vitamin B12 deficiency 12–24 months after surgery if not supplemented with the vitamin [72,73].

In people with vitamin B12 malabsorption, 1000–1500 µg/day of oral vitamin B12 [74,75,76,77] or 1000 µg i.m. B12 every 1–3 months is adequate as a maintenance treatment to keep serum B12 concentrations within the reference range [18,78]. Lowered serum concentrations of vitamin B12 are unlikely to be corrected by using 3–6 µg/day B12 from food supplements or high oral B12 (1000 µg) provided once weekly [30,73,79]. High dose oral and i.m. protocols are likely to be equivalent in correcting serum B12 concentrations [14,15,26]. Available vitamin B12 forms such as cyanocobalamin and methylcobalamin are safe and beneficial [80,81].

Neuropsychiatric conditions are common in patients with B12 deficiency, although the available studies are heterogeneous regarding patient characteristics and the diagnostic tools employed [3,4,5,29,33,82,83,84,85,86,87]. This variability in particular concerns the differentiation between peripheral sensory neuropathy and sensory neuronopathy (gangliopathy) alone or as part of the subacute combined spinal cord degeneration (SCD) syndrome. At least 50% of patients have both manifestations concurrently [24]. Therefore, in patients with sensory symptoms, differential diagnoses for both disorders need to be considered.

The time needed for hematological and neurological symptoms to recover after starting vitamin B12 treatment varies among subjects [14,88], but high-quality follow-up studies are not available. In general, subjective improvement occurs earlier and is more impressive than objective recovery of neurological function [29]. The earliest subjective improvements after B12 treatment occurred for paresthesia and balance, leading to full recovery [87]. Up to 25% of patients have been reported to retain severe neurological symptoms despite the normalization of blood marker levels [8,9]. In approximately 20% of patients with neurological signs and symptoms, recovery may become evident after 3 months of starting the therapy and remain partial [3]. Symptoms of neuropathy mostly improve within several months but may take up to one year for sensory symptoms to resolve after the start of B12 therapy [4]. In patients with SCD, sensory neuronopathy may improve after 7 weeks of B12 therapy (range: 2–32 weeks) [24]. In general, B12 treatment for a minimum of 2 months improves signs and symptoms in all patients with SCD, while Romberg’s sign and mild sensory disturbances in the toes and fingers may persist in some patients after several weeks or months of B12 therapy [7,89]. In a group of deficient patients who were treated and followed up for 2–24 months, the patients with stomatitis showed a complete recovery [4]. In another study, neuropsychological tests were corrected after 6 weeks of multiple i.m. injections of 1000 µg B12 [90]. Functional recovery measured by an activity of daily living score may take as long as 12 months, although single cognitive tests may already show some improvement within 3 months [82].

The duration and severity of pretreatment deficiency symptoms have a clear impact on the time course and degree of recovery after starting B12 therapy [1,86], thus emphasizing the importance of early identification and prevention.

### 3.2. Results of the Delphi Survey

#### 3.2.1. Characteristics of the Survey Panelists

Medical doctors constituted 67% of the 46 panelists in the first round of the survey and 64.4% of the 42 panelists in the second round. The geographical distribution of the panelists in the two survey rounds was as follows: 26.2% were from Asia and the Middle East, 47.6% were from Central and Western Europe, 14.3% were from Eastern Europe, and 11.9% were from North America. The specialties of the 42 panelists in survey 2 were as follows: n = 16 internal medicine, n = 7 neurology, n = 6 other medical specialists, n = 11 researchers in the B12 field, and n = 2 people with medical backgrounds working in B12-related societies or groups (Figure S2).

#### 3.2.2. Delphi Survey Rounds

The questions that received consensus in the first survey (Table S4) were revised and used in the second survey (Table S5). The final consensus statements are summarized in Table 1 and Table 2.

#### 3.2.3. Consensus on the Clinical Practice of Diagnosing Vitamin B12 Deficiency

**Figure 1:**
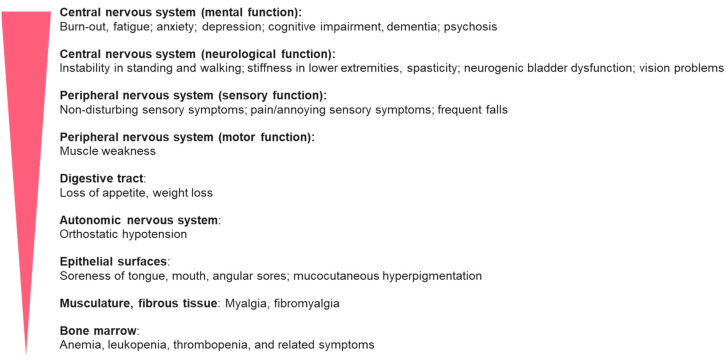
Symptoms of B12 deficiency ordered from the most to the least difficult for being linked to B12 deficiency. Although neuropsychiatric symptoms are more frequent than hematological abnormalities, they are less specific, more demanding to recognize, and thus more difficult to link to B12 deficiency.

The panelists agreed that the delay in the diagnosis of vitamin B12 deficiency may be explained by barriers such as the variability of symptoms, low doctor awareness of the disease, not paying attention to the patient’s symptoms, and limited access to modern laboratory markers (Table 1). There is agreement that some of these barriers can be overcome by educating both doctors and patients. The panelists agreed that symptoms related to the peripheral and central nervous systems are the most common symptoms in patients with clinically manifested vitamin B12 deficiency (Figure S3). At the same time, it was agreed that symptoms affecting the central nervous system (mental function and neurological function), followed by those affecting the peripheral nervous system, are the most difficult symptoms to link to B12 deficiency (Figure 1), suggesting the need for more specific awareness in a primary medical care setting and an improvement in examination options. Moreover, there is agreement that metabolic biomarkers such as methylmalonic acid and homocysteine can be useful in guiding the diagnosis of vitamin B12 deficiency and optimizing and monitoring treatment with vitamin B12 (Table 1). The panelists agreed that the periodic measurement of serum concentrations of vitamin B12 can be helpful in identifying people at risk of deficiency, such as people receiving long-term metformin treatment.

If concentrations of serum vitamin B12 are above the reference range and people have no specific medical conditions, the panelists agreed that inquiring about the usage of supplemental vitamin B12, repeating the measurements of serum B12 after a given time interval, and measuring routine blood markers of renal or liver function or exploring malignancies [91] could explain the cause of high serum B12 (Table 1).

**Figure 2:**
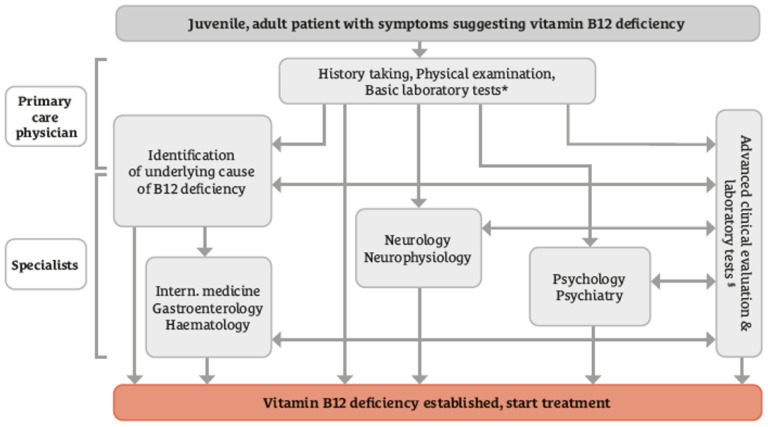
Diagnostic algorithm for vitamin B12 deficiency assessment. The vitamin B12 deficiency diagnostic algorithm was based on a consensus of 42 panelists [mean (95% confidence intervals of the agreement level) = 0.76 (0.61 − 0.88)]. * Full blood cell count and serum B12 concentration; § Serum concentrations of holotranscobalamin, methylmalonic acid, total homocysteine, gastrin, antibodies against parietal cells and/or intrinsic factor and specific tests of the respective specialties. Special diagnostic tests are subject to availability.

In the context of clarifying the cause of vitamin B12 deficiency, the experts agreed that food-cobalamin malabsorption may cause vitamin B12 deficiency in elderly people even if the dietary intake of B12 is adequate and the person has no gastrointestinal disorders. To support identifying the cause of vitamin B12 deficiency, doctors need to inquire whether the person adheres to a diet that is low in foods from animal sources and should ask the person about disease history, regular use of certain medications, and recreational use of nitric oxide. There is agreement that the malabsorption of vitamin B12 can be suspected in people with autoimmune disorders, in patients who have had gastric surgery, in people with a family history of pernicious anemia or with positive test results for serum antibodies against intrinsic factor or parietal cells. Moreover, assessing folate and iron statuses can aid in the differential diagnosis of vitamin B12 deficiency (Table 1). The algorithm for diagnosing vitamin B12 deficiency and its causes was agreed upon among the panelists (Figure 2). In people with symptoms suggesting vitamin B12 deficiency, the general practitioner can collect information on medical history, perform physical examinations, and perform basic laboratory tests such as full blood cell count and serum B12 concentration. Advanced clinical and laboratory markers can be ordered if there are indications (e.g., intrinsic factor antibodies in cases of gastrointestinal symptoms). After the preliminary workup, treatment with B12 can start. Depending on the clinical symptoms, some patients may need referral to other specialties.

#### 3.2.4. Consensus on Clinical Practices of Treatment, Prophylaxis, and Long-Term Management of Vitamin B12 Deficiency

Due to the lack of evidence on the comparative effectiveness and safety of different chemical forms of vitamin B12 (e.g., cyanocobalamin and methylcobalamin), the panelists agreed that evidence from randomized controlled trials is needed. The prophylactic use of vitamin B12 was regarded as necessary for people diagnosed with atrophic gastritis, those with previous bariatric surgery, people at risk of B12 deficiency due to illnesses or medications, people with low or no consumption of animal source foods, and people ever diagnosed with vitamin B12 deficiency when they decided to become pregnant (Table 2).

Regarding the route of administration, it was agreed that the acuity and severity of the symptoms of vitamin B12 deficiency necessitate prioritizing treatment with parenteral vitamin B12. Contraindications to parenteral use, such as in people receiving anticoagulant medication, need to be taken into consideration, and oral supplementation may be preferred in those cases. The decision on the route of administration during long-term treatment needs to balance treatment goals and patient preference. If vitamin B12 treatment failed to alleviate the patient’s symptoms, the panelists agreed that doctors should consider alternative conditions that may explain the symptoms and should reevaluate the appropriateness of the dose of B12 therapy. In nonresponsive cases, doctors can consider using parenteral B12, especially if oral B12 treatment has been used in the past or when serum vitamin B12 is not normalized (Table 2).

The detection and treatment of vitamin B12 deficiency during pregnancy, lactation, and infancy should receive high priority due to the otherwise serious impact on fetal and infant development. Moreover, prophylactic B12 supplementation should be used from prepregnancy until the end of the lactation period in women with previously diagnosed B12 deficiency or who are at risk of vitamin B12 deficiency due for instance to low dietary intake of B12.

Aspects related to the diagnosis and treatment of vitamin B12 deficiency that did not achieve agreement among the panelists (Table S7) are briefly discussed below.

## 4. Discussion

### 4.1. Delphi Consensus

The present work was undertaken with the aim of reviewing the current knowledge related to the diagnosis and treatment of vitamin B12 deficiency. In addition, we sought expert opinion to guide the diagnosis and treatment of vitamin B12 deficiency, especially in areas where there are still heterogeneous views and practices. This study identified controversial topics that need to be investigated in future studies.

Our results suggest that introducing educational elements for healthcare professionals and patients could shorten the path for diagnosing vitamin B12 deficiency and accelerate the initiation of treatment. The need to use different laboratory markers of vitamin B12 to aid in diagnosis stresses the importance of making these markers widely available and affordable, which is not the case in many countries. On the other hand, to avoid costs for the healthcare system, measuring vitamin B12 biomarkers needs to be tailored to the patient’s health condition. Furthermore, the regular measurement of serum vitamin B12 concentrations in all patients at risk (e.g., people using metformin) may cause unnecessary costs in the long term, which needs to be weighed against the costs of vitamin B12 treatment. High serum concentrations of vitamin B12 may be encountered while the clinical picture suggests B12 deficiency. High concentrations of vitamin B12 may be due to underreporting supplement use, autoimmune disorders interfering with the B12 assay, or underlying renal or liver diseases. In addition, some malignancies cause high circulating levels of vitamin B12 and may thereby impact the interpretation of this marker in the context of diagnosing vitamin B12 deficiency [92]. In this case, the concentrations of methylmalonic acid or homocysteine can provide clues on intracellular vitamin B12 status.

The most valuable source for identifying the cause of vitamin B12 deficiency is listening to patients’ complaints, disease history, and clinical examination. Symptoms affecting the nervous system or mental function are more difficult to diagnose and may escape detection due to the low specificity of the symptoms and the limited time available for each patient in primary care settings.

Identifying the cause of vitamin B12 deficiency would enable tailoring the treatment with vitamin B12 (dose, frequency, route of administration, and duration). In people with severe signs and symptoms, the use of injectable forms of vitamin B12 for several weeks is acknowledged, at least in the initial treatment phase. The therapeutic regimen can be revised later when symptoms improve or when patients need to receive vitamin B12 for a longer duration. Prophylactic use of vitamins should receive special attention in at-risk groups and during critical life stages, such as pregnancy and lactation, or in elderly individuals.

### 4.2. Additional Points Raised in the Board Discussion

The board members agreed that in patients with clinically suspected severe B12 deficiency, there must not be any delay in initiating appropriate B12 treatment. Serum specimens for measuring vitamin B12 marker(s) should be collected prior to treatment. Laboratory confirmation of the deficiency can help achieve coverage of B12 treatment costs by health insurance in many countries.

The board discussed that measuring serum B12 concentrations to monitor B12 treatment should be decided on a case-by-case basis (for example, when people show new symptoms, to check compliance with oral treatment, and to check whether B12 was absorbed after oral administration). Although one-third of patients with vitamin B12 deficiency can have normal serum B12 concentrations, measuring serum vitamin B12 is still a convenient screening test when the clinical picture is suggestive of vitamin B12 deficiency. The use of holotranscobalamin as a screening marker for B12 deficiency may increase the cost of the investigations.

The use of vitamin B12 as a prophylactic measure in patients with atrophic gastritis, after bariatric surgery, in vegans, and due to illnesses or medications has reached the consensus of the panelists. The board members highlighted the importance of considering prophylactic vitamin B12 in people using metformin [93] or L-DOPA [94,95] to reduce the possible increased risk of neuropathy due to vitamin B12 deficiency, although this needs to be tested in randomized controlled trials.

There is currently no gold standard for choosing the dose and route of B12 administration. Various protocols have been used successfully. In general, whenever B12 deficiency is clinically manifested or the patient cannot absorb B12, therapeutic doses between 1000 µg/day and 2000 µg/day orally or 1000 µg i.m. (e.g., daily, then weekly, then monthly for maintenance treatment) can be used. The severity of the deficiency symptoms may necessitate choosing injectable forms of B12, at least in the starting phase. The clinical response to B12 treatment in SCD patients is expected to be better when large doses of B12 are administered more frequently than when B12 is administered less frequently [96]. The treatment regimen can be revised later to both fulfill the treatment goals and be convenient for patients.

The scientific board members noted that the duration needed to resolve symptoms of vitamin B12 deficiency can vary. The lack of clinical improvement after 4–8 weeks for anemia and after 6–12 months for neurological signs could suggest that the symptoms are not due to B12 deficiency or that the dose of vitamin B12 or the route of administration needs to be adjusted to the severity of symptoms. Since serum B12 concentrations do not always mirror the clinical picture, this marker is not optimally suited to monitor the effect of treatment, although it can help uncover a lack of patient compliance and malabsorption of oral B12 treatment. An insufficient clinical response to B12 treatment during the first weeks of therapy should not routinely lead to the discontinuation of treatment.

### 4.3. Strengths and Limitations

This study is based on a scoping review that evaluated existing consistent data on practice-relevant topics while identifying areas with an urgent need for consensus where there is still no evidence. The invited scientific board members were not eligible for the surveys, which can ensure survey neutrality. The professionals who participated in the surveys had sufficient diversity regarding their disciplines and countries of practice. Therefore, these consensus points may be generalized to many countries. This study has some limitations, such as the fact that the search was limited to PubMed and to the last 20 years. By limiting the literature search to PubMed, we might have missed studies from parts of the world where vitamin B12 deficiency could be endemic due to economic, cultural, or dietary/religious reasons. The healthcare systems vary across countries, and nutritional deficiencies such as folate, iron, and zinc coexist with B12 deficiency in some parts of the world, suggesting that country-specific recommendations might still be useful, especially in settings with low resources.

### 4.4. Conclusions

The present study provides an overview of consistent data on the diagnosis and treatment of vitamin B12 deficiency. In addition, the Delphi study established a robust consensus on various aspects of diagnosing and treating B12 deficiency to support medical practice in areas where there is insufficient evidence. The experts agreed on the need for educational and organizational changes in the current practices. Although clinical symptoms should receive more weight in diagnosing B12 deficiency, the panelists recognized the need to use serum vitamin B12 concentrations as a cost-effective screening marker and the need to additionally measure a metabolic marker to support the diagnosis. The B12 dose and treatment regimens need to be adjusted according to the severity of the symptoms and the cause of the deficiency. Several topics require future in-depth investigations, such as the clinical benefit of using prophylactic B12 supplementation in some groups who are at risk of B12 deficiency due to accompanying diseases or medications.

## Figures and Tables

**Table 1:** Expert consensus was reached after 2 survey cycles on all of the following topics related to the diagnosis of vitamin B12 deficiency and its causes.

Questions			n (Panelists) ^1^	Mean (95% CI) ^2^
**Identification of vitamin B12 deficiency: Challenges, barriers and obstacles**				
1.	The delay in diagnosing B12 deficiency in a significant number of patients may be due to the following factors:			
	Complexity and variability of signs and symptoms of the deficiency that extend across several medical specialization (hematologic, neuropsychiatric, gastroenterological and other manifestations).		42	0.95 (0.84–0.99)
	Doctors may not have sufficient awareness of risk factors for B12 deficiency.		42	0.93 (0.81–0.99)
	Not paying sufficient attention to patients’ complaints which are often vague.		41	0.85 (0.71–0.94)
	High costs and limited availability of advanced laboratory B12 markers such as plasma methylmalonic acid, total homocysteine and holotranscobalamin.		41	0.68 (0.52–0.82)
2.	The following initiatives can reduce the burden of unidentified B12 deficiency:			
	Increase awareness of doctors and medical personnel toward signs and symptoms of B12 deficiency, diagnostic measures and people at risk.		42	100%
	People at risk of B12 deficiency due to their lifestyle, background diseases, or family history of B12 deficiency should regularly receive understandable information from their doctors explaining causes and consequences of B12 deficiency and possible prophylactic measures.		41	0.83 (0.68–0.93)
3.	Signs and symptoms of B12 deficiency may affect multiple organ systems at variable frequency. The crude order of affected systems (highest to lowest prevalence) is shown in Figure S3.		41	0.71 (0.54–0.84)
4.	The most difficult symptoms to link to clinically manifested B12 deficiency are (as ordered from most to least difficult) as shown in Figure 1.		40	0.80 (0.64–0.91)
5.	Clinically manifested B12 deficiency is commonly first identified in primary medical care. Some patients may require referral to a specialist. Referral of patients to gastroenterologists is least frequent compared to referral to neurologists/psychiatrists and hematologists		38	0.71 (0.54–0.85)
6.	Concordance with the diagnostic pathway shown in Figure 2.		42	0.76 (0.61–0.88)
**Biomarkers and their utility in clinical practice**				
7.	Considering the cost‒benefit and the added value of advanced laboratory tests beyond plasma B12 concentrations and blood cell count:			
	Measurement of a metabolic marker such as plasma methylmalonic acid (or total homocysteine if methylmalonic acid is not available) is useful in guiding the diagnosis of B12 deficiency.		41	0.88 (0.74–0.96)
	If available, plasma methylmalonic acid concentration is a useful marker for monitoring the effectiveness of B12 treatment in general.		41	0.76 (0.60–0.88)
	If available, plasma methylmalonic acid concentration is useful in monitoring the success of oral B12 treatment in particular when it is questionable whether the B12 dose is appropriate or people can absorb B12.		39	0.69 (0.52–0.83)
	Plasma methylmalonic acid concentration (or at least total homocysteine) should be made available for all people suspected of having B12 deficiency.		41	0.83 (0.68–0.93)
	Although the metabolic markers (plasma methylmalonic acid and total homocysteine) have some limitations, they can be very helpful when the clinical picture is uncertain.		41	0.88 (0.74–0.96)
8.	Because chronic use of metformin in patients with diabetes is associated with lower plasma concentrations of B12 and linked to the frequency and severity of neuropathy, measurement of B12 status once per year in this group of patients can help detecting a deficiency prior to clinical manifestation.		40	0.83 (0.67–0.93)
9.	If plasma B12 concentrations far above the reference range are encountered in a person without specific medical conditions:			
	Inquire if the person is using any supplemental B12 source (food supplements or OTC).		41	0.98 (0.87–0.999)
	If the person is not using a B12-supplement, repeat plasma B12 test after few months.		40	0.70 (0.53–0.83)
	Rule out disturbed blood count, liver and renal function markers that may explain high plasma B12 levels due to liver or kidney diseases or undiagnosed malignancies.		39	0.85 (0.69–0.94)
**Identifying the cause of vitamin B12 deficiency**				
10.	A holistic approach is deemed necessary for diagnosing B12 deficiency and identifying the cause(s). This includes:			
	In elderly people food-cobalamin malabsorption may cause B12 deficiency even if the intake of B12 from foods is adequate and in the absence of gastrointestinal disorders.		42	0.93 (0.81–0.99)
	To clarify the cause of B12 deficiency, ask the person about:			
		○ practicing a vegan diet, a vegetarian diet, or avoiding animal source foods.	42	0.93 (0.81–0.99)
		○ gastrointestinal problems or previous gastric or intestinal diseases or surgeries.	42	0.95 (0.84–0.99)
		○ regular use of medications (e.g., gastric acid blockers, metformin, L-DOPA).	42	0.98 (0.87–0.999)
		○ recreational use of laughing gas.	40	0.70 (0.53–0.83)
11.	The following conditions may provide clues for B12 deficiency being due to B12 malabsorption:			
	Autoimmune diseases in the person’s medical history (e.g., thyroid dysfunction, T1DM, celiac disease, or rheumatoid arthritis).		39	0.87 (0.73–0.96)
	Gastric surgery in the person’s medical history (due to cancer or for weight loss).		42	0.98 (0.87–0.999)
	Family history of pernicious anemia.		42	100%
	Chronic gastrointestinal conditions.		41	0.93 (0.80–0.98)
	Positive serum antibody results against parietal cells or intrinsic factor.		42	0.88 (0.74–0.96)
12.	In context of the B12 diagnostic work-up, folate and iron status should also be assessed.		42	0.95 (0.84–0.99)

^1^ Total number of the panelists who answered each of the questions. ^2^ Mean percentage and the 95% confidence intervals of the panelists who considered themselves qualified to answer the question and chose “agree” or “strongly agree” to the answer. We considered that a consensus was reached when the lower bound of the 95% confidence interval was 50% or higher. T1DM, type 1 diabetes mellitus.

**Table 2:** Expert consensus was reached after two survey cycles on all of the following topics related to treatment, prophylaxis, and long-term B12 deficiency.

	Question	n (Panelists) ^1^	Mean (95% CI) ^2^
1.	At present, it is unclear whether different forms of B12 differ in their effectiveness or safety. Clinical trials comparing the safety and effectiveness of the commercially available forms are needed.	42	0.88 (0.74–0.96)
2.	Regarding the use of prophylactic B12 supplementation:		
	Patients with atrophic gastritis may benefit from prophylactic B12 supplementation.	41	0.85 (0.71–0.94)
	People at risk of B12 deficiency due to illnesses or medications should be recommended to use prophylactic B12 supplementation.	41	0.85 (0.71–0.94)
	People who underwent bariatric surgery in the past should receive B12 therapy or prophylactic B12 supplementation for long-term.	41	0.90 (0.77–0.97)
	People with low or no consumption of animal source foods should receive prophylactic B12 supplementation.	42	0.83 (0.69–0.93)
	People ever diagnosed with B12 deficiency, should receive prophylactic B12 supplementation when they decide to become pregnant.	39	0.85 (0.69–0.94)
3.	There is no one-size-fits-all regarding the dose of B12, the frequency and the route of B12 therapy in people with B12 deficiency. Regarding the decision on the route of B12 administration:		
	Higher degrees of acuity and severity of symptoms should lead to prioritizing parenteral B12 treatment over oral treatment.	38	0.87 (0.72–0.96)
	Contraindications of intramuscular injections such as concurrent anticoagulant medication can lead to prioritizing oral B12 treatment.	32	0.75 (0.57–0.89)
	The decision on the route of B12 administration should consider patients’ preference that may change during long-term treatment.	40	0.78 (0.62–0.89)
4.	If B12 treatment fails in symptomatic patients, one or more of the following measures are recommended:		
	Consider alternative diagnoses that may explain the patient’s symptoms.	40	0.98 (0.87–0.999)
	Check if the B12 dose was appropriate.	39	0.95 (0.83–0.99)
	Switch to parenteral B12 if oral B12 treatment was used in the past and plasma B12 has not been normalized.	38	0.87 (0.72–0.96)
5.	B12 deficiency during pregnancy, lactation and in infancy needs to be detected and treated as early as possible because of the serious effects of B12 deficiency on fetal and infant development.	38	0.89 (0.75–0.97)
6.	Women with a previously diagnosed B12 deficiency or dietary restriction of animal foods should take prophylactic B12 supplementation from pre-pregnancy to the end of the lactation period.	38	0.92 (0.79–0.98)

^1^ Total number of the panelists who answered each of the questions. ^2^ Mean percentage and the 95% confidence intervals of the panelists who considered themselves qualified to answer the question and chose “agree” or “strongly agree” to the answer. We considered that a consensus was reached when the lower bound of the 95%confidence intervals is 50% or higher.

## Data Availability

The data that support the findings of this study are available from the corresponding author upon reasonable request.

## Supplementary Materials

The following supporting information can be downloaded at: https://www.mdpi.com/article/10.3390/jcm13082176/s1, Figure S1: Study Flow Diagram; Figure S2: The composition of the 42 panelists who took part in both survey rounds; Figure S3: Vitamin B12 deficiency may cause or be associated with signs and symptoms in multiple organ systems. The crude order of affected systems is shown in the figure from highest prevalence to lowest prevalence according to panelists’ votes in survey 1. Table S1: Keywords and filters used in the literature search in PubMed; Table S2: The inclusion and exclusion criteria used in the scoping review; Table S3: Search results, included and excluded studies in the full-text stage, and the reasons for the exclusions; Table S4: Questions, potential answers, and results of survey 1 and the answers that were qualified for the second Delphi round (survey 2) according to the study criteria; Table S5: Questions included in Delphi survey 2 and the results of agreement or disagreement; Table S6: Topics on vitamin B12 deficiency with consistent results across studies in the literature; Table S7: Topics related to the diagnosis and treatment of vitamin B12 deficiency that did not achieve consensus of the panelists according to the study criteria.
