# Supplementary material for: Diagnosis, Treatment and Long-Term Management of Vitamin B12 Deficiency in Adults: A Delphi Expert Consensus
Source: J Clin Med. 2024 Apr 10;13(8):2176. doi: 10.3390/jcm13082176 (PMC11050313; doi:10.3390/jcm13082176)
Supplement: Supplementary file 1 [file jcm-13-02176-s001.zip › Supplemental Tables Revised_Proof.pdf]

Title: Diagnosis, treatment and long-term management of vitamin B12 deficiency in adults: A Delphi expert consensus

Obeid et al.,

Supplemental Tables

**Table S1.** Keywords and filters used in the literature search in PubMed.	
Population	Adults, vitamin B12 deficient, people with neurological disorders caused by B12 deficiency such as subacute combined degeneration, pernicious anemia, peripheral neuropathy, people with diabetes, dementia, elderly people, intrinsic factor antibody, gastritis, macrocytic anemia, anemia, malabsorption, gastric bypass surgery, neurological disorders, nitrous oxide toxicity, spinal cord diseases, proton pump inhibitors
#1	“deficien*”[tiab] OR neuropathy[tiab] OR tingling[tiab] OR pain[tiab] OR vibration[tiab] OR sensation[tiab] OR “peripheral nerve”[tiab] OR “peripheral neuritis”[tiab] OR symptoms[tiab] OR “nitrous oxide”[tiab] OR “degeneration*”[tiab] OR “diagnos*”[tiab] OR marker[tiab] OR “subacute*”[tiab] OR anemia[tiab] OR anemic[tiab] OR anaemia[tiab] OR anaemic[tiab] OR malabsorption[tiab] OR “B12-malabsorption”[tiab] OR “B12-absorption”[tiab] OR “nerve*”[tiab] “megalo*”[tiab] OR “neuro*”[tiab] OR “nervous*”[tiab] OR “diabetes*”[tiab] OR diabetic[tiab]OR dementia[tiab] OR “intrinsic*”[tiab] OR gastritis[tiab] OR “glossitis*”[tiab] OR atrophy[tiab] OR “sensation”[tiab] OR “spin*”[tiab] OR weakness[tiab] OR “gastrointestinal*”[tiab] OR “sore tongue”[tiab] OR “anorexia”[tiab] OR “achlorhydria”[tiab] OR “numbness”[tiab] OR “vibratory sense”[tiab] OR macrocytosis[tiab] OR leukopenia[tiab] OR locomotion[tiab] OR neuromuscular[tiab] OR “gastric cancer”[tiab] OR appendicitis[tiab] OR paraesthesiae[tiab] OR “spinal cord”[tiab] OR “posterior column”[tiab] OR “mental disturbance”[tiab] OR “papillae atrophy”[tiab] OR “weight loss”[tiab] OR “gastric*”[tiab] OR “sore mouth”[tiab] OR cholecystitis[tiab] OR “cerebr*”[tiab] OR vitiligo[tiab] OR “optic atrophy”[tiab] OR hypertension[tiab] OR atherosclerosis[tiab] OR “proton pump inhibitors*”[tiab] OR “gastrectomy”[tiab] OR “Crohn*”[tiab] OR “inflammatory bowel disease”[tiab] OR “metformin”[tiab] OR parkinson[tiab] OR “L-dopa”[tiab] OR “H2-receptor ant*”[tiab] OR “pylori”[tiab] OR infection[tiab] AND "vitamin b 12 deficiency/complications"[MeSH Terms] OR "vitamin b 12 deficiency/diagnosis"[MeSH Terms] OR "vitamin b 12 deficiency/diagnostic imaging"[MeSH Terms] OR "vitamin b 12 deficiency/diet therapy"[MeSH Terms] OR "vitamin b 12 deficiency/drug therapy"[MeSH Terms] OR "vitamin b 12 deficiency/etiology"[MeSH Terms] OR "vitamin b 12 deficiency/mortality"[MeSH Terms] OR "vitamin b 12 deficiency/prevention and control"[MeSH Terms]
Intervention	Cobalamin, vitamin B12, cyano, hxdroxy, adenosyl and methyl-cobalamin, injection, oral, nasal, and B12 biomarkers

#2	cobalamin[tiab] OR “vitamin B12”[tiab] OR B12[tiab] OR “vitamin*”[tiab] OR “cyanocobalamin”[tiab] OR “hydroxycobalamin”[tiab] OR hydroxocobalamin[tiab] OR “methylcobalamin”[tiab] OR intervention[tiab] OR injection[tiab] OR oral[tiab] OR nasal[tiab] OR biomarker[tiab] OR marker[tiab] OR “methylmalonic*”[tiab] OR “blood count”[tiab] OR “blood test”[tiab] OR diagnose[tiab] OR holotranscobalamin[tiab] OR homocysteine[tiab] OR “anti-intrinsic factor”[tiab] OR antibodies[tiab] OR “parietal cells antibody”[tiab]
Comparison	B12 sufficient, healthy
Outcome	Clinical improvement, improvement of pain, general health, side effects, toxicity
#3	“recover*”[tiab] OR safety[tiab] OR “improve*”[tiab] OR clinical[tiab] OR “symptom*”[tiab] OR improved[tiab] OR safety[tiab] OR “side effect*”[tiab] OR remission[tiab] OR relapse[tiab] OR “prevent*”[tiab]
#4	Exclude animal studies, case reports, infants:<br>mouse[tiab] OR mice[tiab] OR rat[tiab] OR rats[tiab] OR murine[tiab] OR “animal*”[tiab] OR “cell culture”[tiab] OR “cell-culture”[tiab] OR “inborn error*”[tiab] OR newborn[tiab] OR “genetic*”[tiab] OR “case report”[tiab] OR “infant*”[tiab] OR children[tiab] OR “case-report*” OR “inherited” [tiab] OR “gene*”[tiab]
Language Filter	Only English language
Time filter	Time limit last 20 years 2003-2023
Search	#1 AND #2 AND #3 NOT #4

**Table S2.** Inclusion and exclusion criteria used in the scoping review.
**Inclusion criteria:** Observational and interventional studies, systematic reviews and meta-analysis were qualified. The study design can be case-control, cohort, cross sectional or interventional. Intervention studies can be randomized, or quasi-randomized, controlled (placebo or any appropriate comparator) or uncontrolled, blinded or open labelled. Studies of any design reporting improvement of clinical symptoms of certain disorders after intervention with B12 were included. Studies on risk groups or diseases consistently showing high prevalence of B12 deficiency were included. Studies on signs and symptoms (e.g. mouth ulceration, bladder dysfunction, etc) that were historically and consistently found in patients with B12 deficiency were included. Randomized controlled trials, single arm studies, studies treating with B12 (both arms received B12) without a control group were included. The studies must have reported at least one of the following: 1- clinical signs and symptoms claimed to be related to B12 deficiency and were treated with vitamin B12 to proof reversibility or a causal relationship; 2- intervention studies using vitamin B12 alone or in combination with drugs (versus the drug alone) or active compounds (except of combination with other B vitamins such as B6, B1 and folic acid) versus without the additional component. The outcome of interventional studies could be improvement in clinical symptoms and/or based on biomarkers; 3- studies measuring biomarkers of vitamin B12 and studying the effect of vitamin B12 on the biomarkers.<br>The clinical symptoms of vitamin B12 deficiency had to be objectively measured (e.g. MRI, electrophysiology, etc.) or assessed using standardized neurological test scores classifying subjective symptoms and/or objective signs of sensorimotor dysfunction, measuring pain, quality of life, cognitive function tests, the presence of anemia (i.e., by measuring hemoglobin), or any other relevant clinical investigations. In interventional studies, the intervention could be with vitamin B12 of at least 50 µg per treatment session (day or week). We assumed 50 µg vitamin B12 to be the minimal effective dose that may cause clinical improvement. Studies using any form of vitamin B12 (cyano, hydroxyl, methyl, adenosyl) and any route of administration (oral, injection, nasal spray, etc.) were eligible. There was no restriction with regard to the duration of treatment. There was no restriction with regard to the characteristics of the populations under investigation, except for age (had to be $\geq$ 14 years). The study participants could have other comorbidities that could be the reason for B12 deficiency such as intestinal disorders, diabetes, renal diseases, and could be using drugs that interfere with vitamin B12. No restriction was applied regarding the characteristics and site of recruitment of the participants (asymptomatic, hospital settings, free living, and outpatients clinics).
**Exclusion criteria:** Animal studies, in-vitro studies, case reports, case series including < 3 cases, letters to the editor, narrative reviews, and reports on inherited causes of vitamin B12 deficiency (inborn errors, late manifestations of inherited B12 metabolic disorders and genetic variants). Studies on acquired B12 deficiency in infants and children were not eligible due to the limited number of studies with purely vitamin B12 deficiency or single B12 treatment. Observational studies showing novel associations between vitamin B12 and some diseases or conditions (e.g. CVDs, restless legs syndrome, etc.), but not showing reversibility after treatment (where no evidence for causality is supported by the data from the study or the literature) were not eligible.<br>Treatment studies should not include a combination of B12 with other active ingredients, because it cannot be determined whether the improvement of symptoms was due to B12. Cost-effectiveness studies were excluded because they were not the focus of this consensus. We excluded studies investigating primarily homocysteine and folate but not focusing on B12, studies on B12-biomarkers only, but not showing clinical symptoms or reversibility, and studies on maternal low vitamin B12 in context of birth outcomes or birth defects. Studies on the effect of maternal B12 deficiency on outcomes in the fetus/newborn were not the subject of the present investigation. The topic of exposure to nitric oxide was addressed in general, but individual studies on nitric oxide abuse were not included.

Table S3 is provided as a separate Excel table

Table S4. Questions, potential answers and results of survey 1 and the answers that qualified for the Delphi survey round 2 according to the study criteria [(1)].

Questions in survey 1	All possible answers in survey 1	The answers that reached agreement [(1)] and were revised and transferred to survey 2
Q1<br>The overall burden of unidentified B12 deficiency is being commonly underrated. On this background, would you suggest one or more of the following initiatives?	• I do not have the expertise to answer this question.<br>• General practitioners and primary health care personnel need to be more aware of patients at risk, symptoms, diagnostic measures, and management of B12 deficiency.<br>• Patients and the public need to be better informed on the nature, causes and consequences of B12 deficiency.<br>• Establish centralized and specialized multidisciplinary health centers for complex diseases.<br>• I would suggest to e.g., foster self-support groups for B12 deficient patients.<br>• Other (please specify) -----------------	• General practitioners and primary health care personnel need to be more aware of patients at risk, symptoms, diagnostic measures and management of B12 deficiency. **0.98 (0.91-0.998)**<br>• Patients and the public need to be better informed on the nature, causes and consequences of B12 deficiency. **0.86 (0.77-0.93)**
Q2<br>In more than 50% of people with B12 deficiency, the diagnosis is delayed by several years. What are the obstacles preventing an earlier diagnosis, especially for primary health care providers?	• I do not have the expertise to answer this question.<br>• Signs and symptoms of B12 deficiency can vary.<br>• There is insufficient time available during primary care consultations.<br>• There is generally insufficient knowledge among doctors about risk factors for B12 deficiency.<br>• The evaluation of neurological and psychiatric features of B12 deficiency is the prominent diagnostic problem for primary health care providers.<br>• In my setting, the costs of diagnosing B12 deficiency are not entirely covered by the health care system.<br>• Other (please specify)-----------------	Signs and symptoms of B12 deficiency can vary. **0.86 (0.77-0.93)**<br><br>There is generally insufficient knowledge among doctors about risk factors for B12 deficiency. **0.93 (0.85-0.97)**

<table>
<tr>
<td>Q3 (2), (3)
In your opinion, which of the following symptoms are most difficult to link to clinically manifested B12 deficiency?</td>
<td>

I do not have the expertise to answer this question.
Symptoms related to anemia (physical tiredness, paleness, tachycardia, dizziness, headache…)
Non-disturbing sensory neurological dysfunction (numbness, minor paresthesia...)
Pain or other unpleasant sensations (burning, pricking, tightness around ankles...)
Instability in standing or walking
Frequent falls
Orthostatic hypotension
Vision problems
Stiffness (spasticity) in legs, spastic gait
Neurogenic bladder dysfunction (urgency, dyssynergia…)
Muscle weakness
Myalgia or fibromyalgia
Soreness of tongue/beefy red tongue and angular lip soreness
Loss of appetite and unintentional weight loss
Depressive symptoms
Burnout and fatigue
Anxiety
Cognitive impairment and dementia
Psychosis
Mucocutaneous changes: Hyperpigmentation of hair, nails, skin and tongue
Other (please specify)-----------------

</td>
<td>
Table 4
Question 9; Which of the following symptoms are most difficult to link to clinically manifested B12 deficiency
<table>
<tr><th>System grouping of symptoms</th><th>% of total 340 votes agree that the following symptoms are most difficult to link to clinically manifested B12 deficiency (ordered from highest to lowest)</th></tr>
<tr><td>Central nervous system>Mental function, cognition and mood>burn-out, fatigue; anxiety; depression; cognitive impairment, dementia; psychosis</td><td>27,4</td></tr>
<tr><td>Central nervous system>Deep sensation, pyramidal tract, spinal tract, vision> instability standing and walking, stiffness, spasticity, neurogenic bladder dysfunction, vision problems</td><td>22,1</td></tr>
<tr><td>Nervous system>Peripheral nerves>Sensory function>Non-disturbing sensory symptoms, Pain/annoying sensory symptoms, frequent falls</td><td>17,9</td></tr>
<tr><td>Nervous system>Peripheral nerves>Motor function>Muscle weakness</td><td>6,5</td></tr>
<tr><td>Digestive tract>Stomach>Gastric function>Loss of appetite, weight loss</td><td>6,2</td></tr>
<tr><td>Nervous system>Autonomic nervous system>Circulatory regulation>Orthostatic hypotension</td><td>6,2</td></tr>
<tr><td>Epithelial surfaces>Tongue, mucous skin>Barrier, protection>Soreness of tongue, mouth & angular sores and Mucocutaneous changes, hyperpigmentation</td><td>5,3</td></tr>
<tr><td>Musculature>Muscles, fibrous tissue>Locomotion, movements>Myalgia, fibromyalgia</td><td>5,3</td></tr>
<tr><td>Blood>Bone marrow>Erythropoesis>symptoms related to anemia</td><td>3,2</td></tr>
</table>
We weighted the results and ranked the symptoms according to the weighted panelist response. We finally used a crude grouping of symptoms and transferred the question to cycle 2.
</td>
</tr>
<tr>
<td>Q4 (2), (3)
In your patients with B12 deficiency how often do you encounter the following symptoms/ conditions?</td>
<td>

Anemia-related symptoms (weakness, paleness, tachycardia, shortness of breath etc.)
Gastric problems
Non-alcoholic fatty liver disease
Hunter’s glossitis and/or cheilitis
Visual problems (decrease of visual acuity)
Deficits of superficial sensation (reduced perception of touch, pressure, pinprick or temperature)
Paresthesias in upper or lower extremities
Pain in upper or lower extremities
Deficits of deep sensation (perception of vibration, joint position sense, loss of balance, instability of standing or gait, ataxia)
Motor weakness (incl. foot drop, stepping gait)

</td>
<td>The results were weighted and the symptoms were ranked. Then, the results were grouped and ranked according to the body system for cycle 2.</td>
</tr>
</table>

	• Muscle pain/ fibromyalgia<br>• Reduced or absent ankle jerks<br>• Lower limb spasticity (leg stiffness, clonus, brisk knee jerks, Babinski's sign)<br>• Orthostatic hypotension<br>• Depressive symptoms<br>• Burnout, fatigue<br>• Anxiety<br>• Cognitive impairment, dementia<br>• Delusions<br>• Mucocutaneous alterations (hyper-pigmentation of hair, nails, skin, tongue)	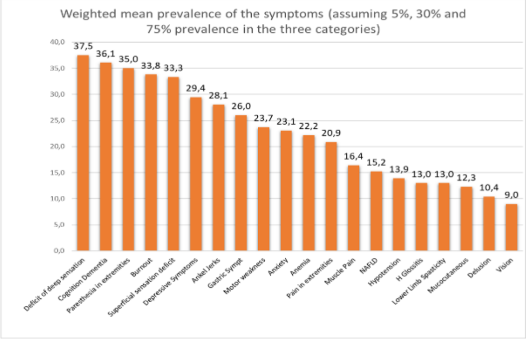<br><br>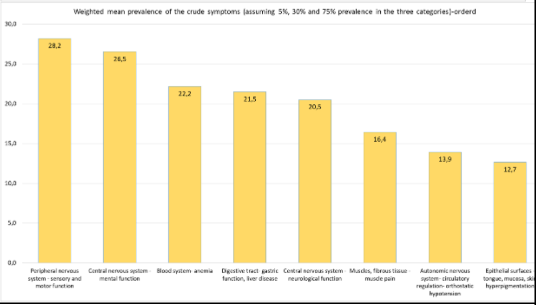
Q5<br>Please indicate whether you concur with this diagnostic pathway (using special diagnostic tests is subject to availability).	• I do not have the expertise to answer this question.<br>• I agree<br>• I do not agree<br>• Some advanced laboratory tests or spinal MRI or neurophysiological measurements are not readily available in my country.<br>• I specifically suggest the following changes (please indicate): -----------------<br>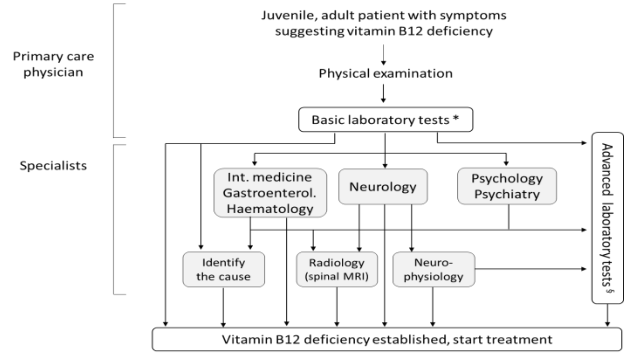 	I agree: **0.73 (0.62-0.82)**<br>17 comments to be taken into consideration whenever possible

Q6<br>Long term use of metformin is associated with lower plasma concentrations of B12 and linked to the frequency and severity of neuropathy. Should B12 status be checked e.g., once per year in people with type 2 diabetes treated with metformin?	• I do not have the expertise to answer this question.<br>• Agree<br>• Neutral<br>• Disagree	I agree: **0.83 (0.74-0.90)**
Q7<br>Plasma B12 concentrations far above the reference range may be encountered during medical checks in people with unspecific symptoms. What is your attitude on this finding?	• I do not have the expertise to answer this question.<br>• I inquire whether the person is using any source of vitamin B12 as food supplement or OTC drugs.<br>• I check liver and renal function markers that may explain high plasma B12.<br>• I rule out clinical conditions that may suggest malignancy (liver, hematological, or others).<br>• I repeat the plasma B12 test after 3-6 months.	• I inquire whether the person is using any source of vitamin B12 as food supplement or OTC drugs. **0.90 (0.81-0.95).**<br>• I check liver and renal function markers that may explain high plasma B12. **0.62 (0.50-0.72)**<br>• I rule out clinical conditions that may suggest malignancy (liver, hematological, or others). **0.62 (0.50-0.72)**<br>• I repeat the plasma B12 test after 3-6 months. **0.72 (0.61-0.81)**
Q8<br>What is your opinion regarding the usefulness of B12 markers in clinical practice?	• I do not have the expertise to answer this question.<br>• For initial diagnosis, I would rely on clinical investigations and disease history and will use B12 related laboratory tests only for orientation/confirmation and occasionally monitoring after treatment.<br>• I would measure plasma B12 concentration for initial diagnosis, taking into consideration that people from a black family background have higher reference intervals for plasma B12.<br>• If possible, I would measure plasma holotranscobalamin in addition to B12.<br>• If possible, I would additionally measure a metabolic marker (methylmalonic acid or total homocysteine) whenever the clinical picture is suggestive of B12 deficiency.<br>• If anemia or neuropsychiatric manifestations are present and plasma B12 concentration is roughly below 300 pmol/L (400 ng/L),<br>I would treat with B12 and use the response of reticulocytes or neuropsychiatric symptoms to treatment within 4 to 8 weeks as diagnostic confirmation of pre-treatment deficiency.	If possible, I would additionally measure a metabolic marker (methylmalonic acid or total homocysteine) whenever the clinical picture is suggestive of B12 deficiency. **0.72 (0.61-0.81)**

	• For monitoring, I would measure plasma B12 only in people who are not on parenteral B12 replacement.	
Q9<br>What is your opinion on the use of disturbed B12 markers (low plasma B12 or holotranscobalamin concentrations optimally combined with elevated methylmalonic acid or homocysteine) to identify B12 deficiency and guide the decision on prophylaxis or treatment of B12 deficiency?	• I do not have the expertise to answer this question.<br>• Disturbed B12 markers in otherwise healthy person are a sufficient criterion to recommend B12 supplementation.<br>• Disturbed B12 markers are not sufficient to diagnose B12 deficiency unless combined with anemia and/or neuropsychiatric symptoms.<br>• Disturbed B12 markers are sufficient to diagnose B12 deficiency in persons at-risk of deficiency because of family history of pernicious anemia or conditions like atrophic gastritis, metformin or L-dopa-medication, gastrectomy, or chronic intestinal diseases e.g., Crohn's disease.<br>• Disturbed B12 markers are sufficient to initiate B12 supplementation in persons with conditions such as dementia, mild cognitive dysfunction, psychiatric symptoms, or neuropathy that may be caused or worsened by B12 deficiency.	• Disturbed B12 markers in otherwise healthy person are a sufficient criterion to recommend B12 supplementation. **0.70 (0.59-0.80)**<br>• Disturbed B12 markers are sufficient to diagnose B12 deficiency in persons at-risk of deficiency because of family history of pernicious anemia or conditions like atrophic gastritis, metformin or L-dopa-medication, gastrectomy, or chronic intestinal diseases e.g., Crohn's disease. **0.70 (0.59-0.80)**<br>• Disturbed B12 markers are sufficient to initiate B12 supplementation in persons with conditions such as dementia, mild cognitive dysfunction, psychiatric symptoms, or neuropathy that may be caused or worsened by B12 deficiency. **0.70 (0.59-0.80)**
Q10<br>Whenever B12 deficiency is diagnosed, the search for the cause of the deficiency should guide the treatment in the long term. Do you agree with one or more of the following measures?	• I do not have the expertise to answer this question.<br>• Ask the person about lifestyle factors that could explain B12 deficiency such as vegetarian/vegan diet.<br>• Ask about family history of B12 deficiency or pernicious anemia.<br>• Ask the person about gastrointestinal complaints, previously diagnosed atrophic gastritis or H. pylori infection, or drugs (e.g. gastric acid blockers, metformin, L-dopa, colchicine, anticonvulsants).<br>• Measure intrinsic factor antibodies first in serum before measuring parietal cell antibodies (if available).<br>• Measure parietal cell antibodies in serum if intrinsic factor antibodies are negative (if available).<br>• Measure serum gastrin (if available).<br>• Measure serum anti-H. pylori antibodies (if available).<br>• If any of the laboratory parameters detailed above is pathological, a gastroscopy is indicated to rule out gastric cancer.<br>• Recognize that elderly people (> 65 years) may have food-cobalamin malabsorption without obvious clinical symptoms that can be linked to B12 deficiency.	• Ask the person about lifestyle factors that could explain B12 deficiency such as vegetarian/vegan diet. **1.00 (0.95-1.00)**<br>• Ask about family history of B12 deficiency or pernicious anemia. **0.91 (0.82-0.96)**<br>• Ask the person about gastrointestinal complaints, previously diagnosed atrophic gastritis or H. pylori infection, or drugs (e.g. gastric acid blockers, metformin, L-dopa, colchicine, anticonvulsants). **0.95 (0.88-0.99)**<br>• Recognize that elderly people (> 65 years) may have food-cobalamin malabsorption without obvious clinical symptoms that can be linked to B12 deficiency. **0.79 (0.69-0.87)**

Q11<br>In your opinion, which signs, symptoms, and tests suggest that a person diagnosed with B12 deficiency may have B12 malabsorption?	• I do not have the expertise to answer this question.<br>• Age > 65 years even without other illnesses.<br>• Indigestion, other gastrointestinal symptoms, or prolonged use of acid blockers.<br>• Elevated serum gastrin.<br>• Positive serum anti-parietal cell antibodies or anti-intrinsic factor antibodies.<br>• Positive serum anti-H. pylori antibodies.<br>• History of autoimmune diseases such as thyroid dysfunction, T1DM, celiac disease, or rheumatoid arthritis in context of symptoms such as neuropsychiatric disorders and/or anemia.<br>• Other (please specify)<br>• I do not have the expertise to answer this question.	• Age > 65 years even without other illnesses. **0.72 (0.61-0.81)**<br>• Indigestion, other gastrointestinal symptoms, or prolonged use of acid blockers. **0.81 (0.72-0.89)**<br>• Positive serum anti-parietal cell antibodies or anti-intrinsic factor antibodies. **0.81 (0.72-0.89)**<br>• History of autoimmune diseases such as thyroid dysfunction, T1DM, celiac disease, or rheumatoid arthritis in context of symptoms such as neuropsychiatric disorders and/or anemia. **0.72 (0.61-0.81)**
Q12<br>In people with malabsorption syndromes, vitamin B12 deficiency may be associated with folate and iron deficiency. Choose one or more of the following statements that apply to non-pregnant individuals.	• I do not have the expertise to answer this question.<br>• In conjunction with the B12 diagnostic work-up, folate and iron status should also be assessed.<br>• Folate status does not need to be evaluated in countries applying mandatory fortification of foods with folic acid.<br>• B12 supplementation should be optimally added to any folic acid therapy without the need to measure B12.	In conjunction with the B12 diagnostic work-up, folate and iron status should also be assessed. **0.98 (0.91-0.100)**
Q13<br>In primary medical care, how often would it be necessary to refer a patient with anemia or neuropsychiatric symptoms of B12 deficiency to a hematologist?	• I do not have the expertise to answer this question.<br>• <10% of patients<br>• 10- 30% of patients<br>• > 30% of patients	< 10% of the patients. **0.67 (0.54-0.78)**
Q14<br>In primary medical care, how often would it be necessary to refer a patient with anemia or neuropsychiatric symptoms of B12 deficiency to a neurologists and psychiatrist?	• I do not have the expertise to answer this question.<br>• <10% of patients<br>• 10- 30% of patients<br>• > 30% of patients	No answer reached the agreement cutoff level
Q15<br>In primary medical care, how often would it be necessary to refer a patient with anemia or neuropsychiatric symptoms of	• I do not have the expertise to answer this question.<br>• <10% of patients<br>• 10- 30% of patients<br>• > 30% of patients	No answer reached the agreement cutoff level

B12 deficiency to a gastroenterologist?		
Q16<br>In patients with B12 deficiency, polyneuropathy as well as subacute combined degeneration of the spinal cord (SCD) may similarly cause sensory deficits, paresthesia and/or instability of stand and gait. Which statements best describe your attitude?	• I do not have the expertise to answer this question.<br>• I would be able to separate polyneuropathy from SCD in most patients based on history and clinical examination.<br>• I would not care too much about separating SCD from polyneuropathy as there is a fair chance that both may improve within several weeks on B12 therapy.<br>• I know that the differential diagnoses of polyneuropathy differ from those of SCD. Therefore, I would refer the patient to neurological/neurophysiological evaluation.<br>• If available, I would refer the patient for the assessment of SCD myelopathy by spinal MRI.	No answer reached the agreement cutoff level
Q17<br>In which of the following risk groups of asymptomatic people would you recommend prophylactic B12 supplementation?	• I do not have the expertise to answer this question.<br>• People with diabetes in general.<br>• People with diabetes only if they are on metformin treatment.<br>• People with atrophic gastritis.<br>• People using long-term PPI or acid blockers.<br>• People with Parkinson's disease on L-dopa.<br>• People with mental illnesses.<br>• Users of anticonvulsive drugs.<br>• People with renal dysfunction.<br>• Users of oral contraceptive pills.<br>• People with low meat intake or a history of B12 deficiency whenever they plan to become pregnant.<br>• Other (please specify)…………….	People with atrophic gastritis. **0.82 (0.72-0.90)**<br><br>People with low meat intake or a history of B12 deficiency whenever they plan to become pregnant. **0.80 (0.70-0.88)**<br><br>9 comments from the panelists
Q18<br>Clinically relevant vitamin B12 deficiency (e.g., anemia and/or neurological symptoms) can develop after 1 to 2 years of various bariatric surgeries. Do you agree with one or more of the following statements?	• I do not have the expertise to answer this question.<br>• Prophylactic B12 supplementation should start before or directly after bariatric surgery, independent of plasma B12 concentrations.<br>• B12 supplementation should be recommended for life in this group.<br>• B12 therapy is not indicated unless plasma B12 concentrations are lowered (or methylmalonic acid or total homocysteine is elevated), or clinical symptoms (anemia or neuropsychiatric) are manifested.<br>• Treatment monitoring should include measurements of plasma B12 at first follow visit and later as needed.<br>• An oral B12 dose between 1 to 2 mg/d is most likely sufficient to ensure passive B12 absorption after bariatric surgeries.	B12 supplementation should be recommended for life in this group. **0.67 (0.55-0.77)**
Q19	• I do not have the expertise to answer this question.<br>• Yes, all forms are equally effective.	I do not think that all forms are equally effective because: **0.66 (0.53-0.77)**

At present, there are no published clinical trials comparing the effectiveness and safety of different forms of B12 (hydroxo-, cyano-, methyl-, and adenosyl-cobalamin) in improving clinical outcomes of B12 deficiency. Do you think that all forms of oral B12 are equally effective at therapeutic doses (e.g., 1 mg)?	• I do not think that all forms are equally effective because:-----------------	21 comments submitted to be considered in the revised question
Q20<br>How would you decide on the route of B12 therapy in B12 deficiency?	• I do not have the expertise to answer this question.<br>• I generally prefer parenteral treatment.<br>• I maintain parenteral B12 application by medical personnel to ensure compliance and effectiveness.<br>• I prefer parenteral treatment during the first month and then offer the patient to shift to oral therapy.<br>• I generally prefer high-dose (1 to 2 mg) oral treatment.<br>• Oral treatment is my first choice in patients taking anticoagulants.<br>• High-dose oral B12 medication is not licensed in my country, therefore I need to stick to parenteral B12 therapy.<br>• I would discuss the B12 therapy protocol with the patient for optimal compliance.<br>• I would decide solely based on the clinical manifestations whether to use parenteral or oral therapy.	No answer reached the agreement cutoff level
Q21<br>Most people with B12 deficiency will need replacement therapy with B12 for many years. What is your view on continuous high-dose oral B12 replacement?	• I do not have the expertise to answer this question.<br>• Monitoring patients with long-term B12 replacement mainly relies on repetitive clinical assessments.<br>• I measure plasma B12 concentrations (every 6-12 months) to investigate whether oral B12 is absorbed and to verify the patient's compliance with the therapeutic schedule.<br>• There is no need to re-check B12 concentrations on a regular basis if people are satisfied with treatment and if there are no new clinical symptoms.<br>• I measure plasma B12 concentrations only at first-follow up appointment after starting B12 replacement.<br>• I am generally not in favour of long-term oral B12 replacement.<br>• I am generally reluctant to prescribe long-term B12 treatment because of (please specify):-----------------	No answer reached the agreement cutoff level

Q22<br>Which approach would you follow if the clinical symptoms of a person do not improve after 4-8 weeks of replacement therapy with B12 (either oral or parenteral)?	• I do not have the expertise to answer this question.<br>• I would explore causes other than B12 deficiency.<br>• I will re-investigate plasma B12 concentrations to verify if they are increased by treatment.<br>• I will additionally measure methylmalonic acid or homocysteine concentrations if available.<br>• I will continue the treatment for further 4-6 months, because many symptoms may take longer to improve.<br>• If anemia is not improved, I would check iron and folate deficiency as additional causes of anemia.<br>• I will consider higher doses of B12 or provide B12 as parenteral instead of oral therapy.	I would explore causes other than B12 deficiency. **0.71 (0.60-0.80)**
Q23B12 deficiency manifested for the first time during pregnancy and lactation may cause severe neurological and hematological damage to the fetus and breast-fed infants, respectively. Which of the following statements reflect your view?	• I do not have the expertise to answer this question.<br>• It is necessary to examine pregnant women for signs and symptoms of B12 deficiency as early as possible.<br>• If B12 deficiency is incidentally detected in a breast-feeding woman, the B12 status must be also investigated in the baby.<br>• If B12 deficiency is detected in an exclusively breast-fed infant, the B12 status must be also investigated in the mother.<br>• B12 should anyway be added to all antenatal supplements.<br>• Plasma B12 concentrations could be transiently low during pregnancy, but not reflecting a deficiency state. In this case, restricted B12 dietary intake, the presence of clinical symptoms such as anemia, and elevated plasma concentrations of methylmalonic acid or total homocysteine can guide the decision of whether the woman should receive additional B12 supplements.<br>• Intake of cyanocobalamin during pregnancy and lactation is safe and does not harm the fetus and the infant.	• It is necessary to examine pregnant women for signs and symptoms of B12 deficiency as early as possible. **0.68 (0.54-0.80)**<br>• If B12 deficiency is incidentally detected in a breast-feeding woman, the B12 status must be also investigated in the baby. **0.79 (0.65-0.88)**<br>• If B12 deficiency is detected in an exclusively breast-fed infant, the B12 status must be also investigated in the mother**. 0.79 (0.65-0.88)**
Q24<br>Laughing gas (nitrous oxide, N2O) may lead to acute or subacute symptoms of B12 deficiency. Which of the following statements reflect your view?	• I have no personal experience in this field.<br>• There is insufficient awareness regarding B12 deprivation caused by N2O.<br>• I realize that macrocytic anemia is not a useful early indicator of B12 deficiency in N2O poisoning, while homocysteine or methylmalonic acid are abnormal early in > 90% of the patients.	There is insufficient awareness regarding B12 deprivation caused by N2O. **0.96 (0.86-1.00)**

(1) In survey 1, answers reached agreement according to the following definition (except for Q3 and Q4 above): first, we calculated the mean and (80% confidence intervals, CIs) of the proportion of panelists who chose a specific answer. Second, when the lower bound of the 80% CIs was ≥ 50%, the corresponding question (sub-question) was transferred to the next cycle of the survey (survey 2).
(2) Only physicians were eligible to answer Q3 and Q4.

(3) Answers to Q3 and Q4 were separately analyzed and weighted to obtain a ranked order of the possibilities.

**Table S5.** Questions included in the Delphi survey 2 and the results of agreement or disagreement [1].		
**I- Questions related to diagnosis of B12 deficiency and its causes**		
	Questions and answers	Mean percentage of the panelists who agreed with the answer and the 95% confidence intervals of the mean (level of agreement)
**1.**	The delay in diagnosing B12 deficiency in a significant number of patients may be due to several factors. Please indicate your agreement/disagreement with each factor mentioned in <u>the questions 1 to 4:</u><br>Complexity and variability of signs and symptoms of the deficiency that extend across several medical specialization (hematologic, neuropsychiatric, gastroenterological and other manifestations).	0.95 (0.84 − 0.99)
**2.**	Doctors may not have sufficient awareness of risk factors for B12 deficiency.	0.93 (0.81 − 0.99)
**3.**	Not paying sufficient attention to patients' complaints which are often vague.	0.85 (0.71 − 0.94)
**4.**	High costs and limited availability of advanced laboratory B12 markers such as plasma methylmalonic acid, total homocysteine and holotranscobalamin.	0.68 (0.52 − 0.82)
**5.**	The following initiatives can reduce the burden of unidentified B12 deficiency. Please indicate your agreement/disagreement for each measure detailed in <u>questions 5 and 6:</u><br>Increase awareness of doctors and medical personnel towards signs and symptoms of B12 deficiency, diagnostic measures and people at risk.	100%
**6.**	People at risk of B12 deficiency due to their lifestyle, background diseases, or family history of B12 deficiency should regularly receive understandable information from their doctors explaining causes and consequences of B12 deficiency and possible prophylactic measures.	0.83 (0.68 − 0.93)
**7.**	Do you agree on the most difficult symptoms to link to clinically manifested B12 deficiency (ordered from most to least difficult according to the panelists' voting in round 1)?<br>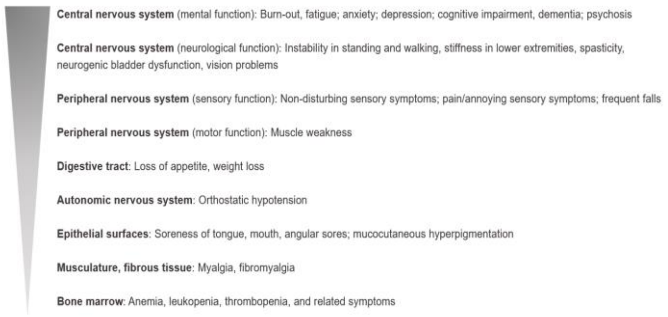	0.80 (0.64 − 0.91)

**8.**	Signs and symptoms of B12 deficiency may affect multiple organ systems at variable frequency. Do you agree with the following crude order of affected systems (highest to lowest prevalence according to panelists' votes in survey 1):<br>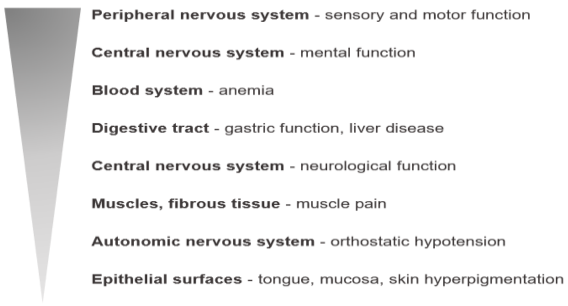 	0.71 (0.54 − 0.84)
**9.**	To which extent do you agree or disagree with the following statement?<br>Because chronic use of metformin in patients with diabetes is associated with lower plasma concentrations of B12 and linked to the frequency and severity of neuropathy, measurement of B12 status once per year in this group of patients can help detecting a deficiency prior to clinical manifestation.	0.83 (0.67 − 0.93)
**10.**	What would you suggest if you encounter plasma B12 concentrations far above the reference range in a person without specific medical conditions? Please indicate your agreement/disagreement with each measure detailed in <u>questions 10 to 12:</u><br>Inquire if the person is using any supplemental B12 source (food supplements or OTC).	0.98 (0.87− 0.999)
**11.**	If the person is not using a B12 supplement, repeat plasma B12 test after few months.	0.70 (0.53 − 0.83)
**12.**	Rule out disturbed blood count, liver and renal function markers that may explain high plasma B12 levels due to liver or kidney diseases or undiagnosed malignancies.	0.85 (0.69 − 0.94)
**13.**	Considering the cost-benefit and the added value of advanced laboratory tests beyond plasma B12 concentrations and blood cell count, please indicate your agreement/disagreement with the statements detailed in <u>13 to 18:</u><br>Measurement of a metabolic marker such as plasma methylmalonic acid (or total homocysteine if methylmalonic acid is not available) is useful in guiding the diagnosis of B12 deficiency.	0.88 (0.74 − 0.96)
**14.**	If available, plasma methylmalonic acid concentration is a useful marker for monitoring the effectiveness of B12 treatment in general.	0.76 (0.60 − 0.88)
**15.**	If available, plasma methylmalonic acid concentration is useful in monitoring the success of oral B12 treatment in particular when it is questionable whether the B12 dose is appropriate or people can absorb B12.	0.69 (0.52 − 0.83)
**16.**	Plasma methylmalonic acid concentration (or at least total homocysteine) should be made available for all people suspected of having B12 deficiency.	0.83 (0.68 − 0.93)

**17.**	Although the metabolic markers (plasma methylmalonic acid and total homocysteine) have some limitations, they can be very helpful when the clinical picture is uncertain.	0.88 (0.74 − 0.96)
**18.**	Measuring plasma holotranscobalamin concentrations as a first line or screening marker should replace measuring plasma vitamin B12 whenever it is available.	**0.53 (0.36 − 0.68)**
**19.**	A holistic approach is deemed necessary for diagnosing B12 deficiency and identifying the cause(s). Please indicate your agreement/disagreement with each measure detailed in 19 to 23:<br>In elderly people food-cobalamin malabsorption may cause B12 deficiency even if the intake of B12 from foods is adequate and in the absence of gastrointestinal disorders.	0.93 (0.81 − 0.99)
**20.**	To clarify the cause of B12 deficiency, ask the person about practicing a vegan diet, a vegetarian diet, or avoiding animal source foods.	0.93 (0.81 − 0.99)
**21.**	To clarify the cause of B12 deficiency, ask the person about gastrointestinal problems or previous gastric or intestinal diseases or surgeries.	0.95 (0.84 − 0.99)
**22.**	To clarify the cause of B12 deficiency, ask the person about regular use of medications (e.g., gastric acid blockers, metformin, L-dopa, ...).	0.98 (0.87 − 0.999)
**23.**	To clarify the cause of B12 deficiency, ask the person about recreational use of laughing gas.	0.70 (0.53 − 0.83)
**24.**	The following conditions may provide clues for B12 deficiency being due to B12 malabsorption. Please indicate your agreement/disagreement for each condition detailed in questions 24 to 28:<br>Autoimmune diseases in the person's medical history (e.g., thyroid dysfunction, T1DM, celiac disease, or rheumatoid arthritis).	0.87 (0.73 − 0.96)
**25.**	Gastric surgery in the person's medical history (due to cancer or for weight loss).	0.98 (0.87 − 0.999)
**26.**	Family history of pernicious anemia.	100%
**27.**	Chronic gastroenteric conditions.	0.93 (0.80 − 0.98)
**28.**	Positive serum antibody results against parietal cells or intrinsic factor.	0.88 (0.74 − 0.96)
**29.**	Please indicate whether you agree or disagree with the following statement:<br>In context of the B12 diagnostic work-up, folate and iron status should also be assessed.	0.95 (0.84 − 0.99)
**30.**	Clinically manifested B12 deficiency is commonly first identified in primary medical care. Some patients may require referral to a specialist. The previous survey suggested the following order of specialist referrals. Please indicate whether you agree or disagree with the order as shown in Questions 30 to 32:<br>Referral of patients to neurologists or psychiatrists (most frequent)	0.58 (0.41 − 0.73)
**31.**	Referral of patients to hematologists is less frequent than referral to neurologists or neuropsychiatrists and more frequent than referral to gastroenterologists	0.61 (0.43 − 0.76)
**32.**	Referral of patients to gastroenterologists is least frequent compared to referral to neurologists/psychiatrists and hematologists	0.71 (0.54 − 0.85)

**33.**	Please indicate whether you concur with this diagnostic pathway (special diagnostic tests are subject to availability).<br><br>*Full blood cell count and plasma B12 concentration<br>§ Plasma concentrations of holotranscobalamin, methylmalonic acid, total homocysteine, gastrin, antibodies against parietal cells and/or intrinsic factor and specific tests of respective specialties	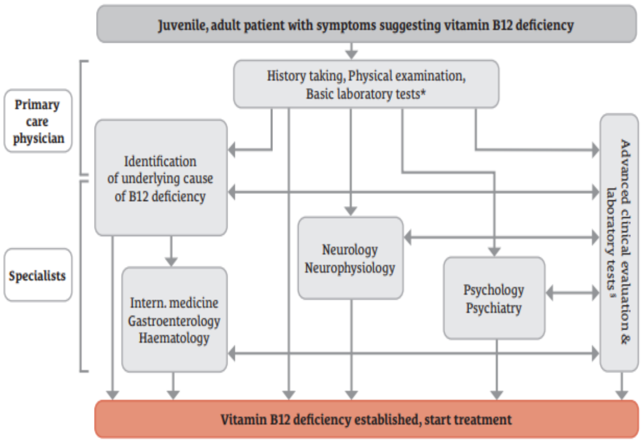	0.76 (0.61 − 0.88)
**II- Questions related to treatment, prophylaxis and long term management**			
**34.**	Regarding the use of **prophylactic** B12 supplementation, please indicate your agreement/disagreement with each of the statements detailed in questions 34 to 38:<br><br>Patients with atrophic gastritis may benefit from prophylactic B12 supplementation.		0.85 (0.71− 0.94)
**35.**	People at risk of B12 deficiency due to illnesses or medications should be recommended to use prophylactic B12 supplementation.		0.85 (0.71 − 0.94)
**36.**	People who underwent bariatric surgery in the past should receive B12 therapy or prophylactic B12 supplementation for long-term.		0.90 (0.77 − 0.97)
**37.**	People ever diagnosed with B12 deficiency, should receive prophylactic B12 supplementation when they decide to become pregnant.		0.85 (0.69 − 0.94)
**38.**	People with low or no consumption of animal source foods should receive prophylactic B12 supplementation.		0.83 (0.69 − 0.93)
**39.**	At present, it is unclear whether different forms of B12 differ in their effectiveness or safety. Do you agree that: Clinical trials comparing the safety and effectiveness of the commercially available forms are needed.		0.88 (0.74 − 0.96)
**40.**	There is no one-size-fits-all regarding the dose of B12, the frequency and the route of B12 therapy in people with B12 deficiency. Regarding the decision on the route of B12 administration, please indicate your agreement/disagreement with each statement in 40 to 43:<br><br>Higher degrees of acuity and severity of symptoms should lead to prioritizing parenteral B12 treatment over oral treatment.		0.87 (0.72 − 0.96)
**41.**	Contraindications of intramuscular injections such as concurrent anticoagulant medication can lead to prioritizing oral B12 treatment.		0.75 (0.57 − 0.89)
**42.**	The decision on the route of B12 administration should consider patients' preference that may change during long-term treatment.		0.78 (0.62 − 0.89)

**43.**	The choice of the route of B12 administration is restricted by economical or regulatory factors and availability of pharmaceutical B12 products.	0.63 (0.46 − 0.78)
**44.**	If B12 treatment fails in symptomatic patients, one or more of the following measures are recommended. Please indicate your agreement/disagreement with each measure detailed in 44 to 46:<br>Consider alternative diagnoses that may explain the patient's symptoms.	0.98 (0.87 − 0.999)
**45.**	Check if the B12 dose was appropriate.	0.95 (0.83 − 0.99)
**46.**	Switch to parenteral B12 treatment if oral treatment was used in the past and if serum B12 has not been normalized.	0.87 (0.72 − 0.96)
**47.**	Do you agree/disagree with the following statement?<br>B12 deficiency during pregnancy, lactation and in infancy needs to be detected and treated as early as possible because of the serious effects of B12 deficiency on fetal and infant development.	0.89 (0.75 − 0.97)
**48.**	Do you agree/disagree with the following statement?<br>Women with previously diagnosed B12 deficiency or dietary restriction of animal foods should take prophylactic B12 supplementation from pre-pregnancy to the end of the lactation period.	0.92 (0.79 − 0.98)

(1) Survey 2 used a 5-points Likert scale (strongly agree, agree, neutral, disagree, and strongly disagree). The percentage of people who selected agree or strongly agree and the 95% confidence intervals (CI) were calculated. The question was considered to reach agreement when the lower bound of the 95%CI of the proportion of the panelists who chose the answer was ≥ 50%.
The grey color is used to show questions that did not reach a consensus.

Table S6. Topics on vitamin B12 deficiency with consistent results across studies in the literature.	
**Topic**	**Consistent results in the literature since 2003**
Vitamin B12 markers in relation to clinically manifested B12 deficiency	Most studies define vitamin B12 deficiency based on serum or plasma concentrations of vitamin B12. Low serum B12 is not present in all people with neurological or hematological manifestations of B12 deficiency [1]. 30%-40% of patients with peripheral neuropathy [2] or subacute combined degeneration of the nerve (SCD) [3,4] may have normal plasma B12 concentrations. Low concentrations of serum B12 are not associated with the severity of the clinical symptoms in patients with SCD [5].<br>Both plasma/serum B12 and methylmalonic acid do not predict the neurological symptoms [6] or the response of neuropathy to treatment [7,8]. Anemia or macrocytosis is not related to the severity of the symptoms of SCD [5,9,10] or to the functional disorder scale [3]. Anemia must not be present in people with neurological symptoms related to B12 deficiency [11].
Diabetes and metformin use	Using metformin is associated with 60-120 pmol/L lower plasma vitamin B12 concentrations [12-18]. There appears to be a dose-response relationship between metformin dose and duration of use of metformin and lowering plasma vitamin B12 [19-21]. The effect of metformin on B12 concentrations is seen after 3 months of starting the drug [19]. Low vitamin B12 in metformin users is associated with elevated plasma homocysteine [22], higher prevalence of neuropathy [23], and worse neuropathy scores [24]. Vitamin B12 supplementation can increase B12 concentrations in people with diabetes who are using metformin [19,25].
The elderly	Vitamin B12 deficiency is common in elderly people [26,27] and may be explained by food malabsorption disorders [28,29]. The associations with different diseases such as anemia [30-32], frailty, sarcopenia [27,33] are inconsistent.
Causes of B12 deficiency due to malabsorption	Macrocytosis can be present in approximately 30% of patients with severe vitamin B12 deficiency [34]. Antibodies against parietal cells or intrinsic factor are found in 58% and 14%, respectively, of people with clinically manifested B12 deficiency [34-36].<br>Pernicious anemia is associated with positive parietal cell antibodies [37] and multiple nutrients deficiency [38] and often occurs parallel to other autoimmune disorders [39] and in persons with first-degree relatives affected with pernicious anemia [37]. Measuring serum gastrin and gastric auto-antibodies may uncover the primary cause of B12 deficiency [40]. Symptoms of autoimmune gastritis are abdominal bloating with or without nausea, constipation, and abdominal pain in 47% of the patients [37].
Gastrointestinal disorders and surgeries	Low serum B12 concentrations are common in patients with atrophic gastritis [41,42], Crohn’s disease [43-45], celiac disease [46], H.pylori infection [47], people using proton pump inhibitors [48], and after gastrectomy [49-53]. Up to 70% of patients with gastrectomy may develop vitamin B12 deficiency after 12-24 months of the gastric surgery if they were not receiving supplemental source of the vitamin in a sufficient dose and frequency [54,55].
B12 treatment in gastrectomy or bariatric surgery	Most available treatment studies investigated the effect of vitamin B12 (various doses, route of administration and frequencies) on plasma B12 concentrations (as an outcome). Oral vitamin B12 supplementation (3-6 µg/d or 1000 µg/ week) is not sufficient to prevent vitamin B12 depletion or deficiency in the majority of patients with disorders causing B12 malabsorption [55-57]. Supplementing 1000 µg/d or 1500 µg/d in patients with total gastrectomy after gastric cancer or those with gastric bypass surgery was more likely to be associated with improved serum B12 compared to doses between 350 µg/d and 500 µg/d [58-61]. Also 1000 µg i.m. B12 every three months was likely to maintain normal serum B12 concentrations after a bariatric surgery [62].

	A dose of 500 µg/d was associated with lower prevalence of vitamin B12 deficiency in patients after 3 years of bariatric surgery [50]. The optimal treatment modality after a bariatric surgery is inconclusive. Studies using high oral dose of B12 on daily basis or i.m. repeated several times were able to correct B12 status during the follow up [57,63-65]. The success of the treatment may depend on vitamin B12 status before the surgery and on the size and location of the resection. Both oral and i.m. protocols are likely to correct plasma B12 concentrations [65]. There are no comparison studies between different B12 forms (cyano- and methylcobalamin). It seems that both forms are safe and beneficial [66,67]. Oral B12 supplementation for 3-4 months was effective in correcting or raising plasma B12 concentrations [67,68]. However, clinical improvement may not be achieved as fast as the correction of plasma B12 concentrations and plasma B12 concentrations are not likely to accurately reflect clinical response.
Neuropsychatric components of vitamin B12 deficiency	Several clinical reports emphasized the frequency of neuropsychiatric dysfunction in patients with B12 deficiency. However, the studies were heterogeneous with regard to the study populations and clinical investigation tools [11,35,36,69-76]. In general, the high percentages of abnormal results with somatosensory evoked potentials (SEP; 80-85%) [36,71], followed by magnetic evoked potentials (MEP) and visually evoked potentials (VEP) [36,71] reflects the leading role of the central nervous system pathology. Therefore, simple peripheral nerve conduction studies would be abnormal in more than half the patients, and, would additionally have the advantage of discriminating peripheral neuropathy from myelopathy.<br>The treatment regime started with daily injections of 1000 µg of cyanocobalamin for e.g. 1-2 weeks, followed by roughly one month of weekly 1000 µg injections, and finally one injection per month without fixed time limits. Follow-up examinations started 2 to 3 months after initiation of treatment in most studies, extending up to more than 3 years. Serum B12 concentrations and hematological markers of B12 function normalized in almost all patients within 2 to 3 months, while neurological and psychiatric impairment took longer to respond. In general, subjective improvement occurred earlier and was more impressive than the objective recovery of neurological function [75]. Thus, functional recovery measured by an activity of daily living score may take as long as 12 months, although single cognitive tests may already show some improvement within 3 months [69]. The earliest subjective improvements occurred for paresthesias and balance, leading to full recovery in single studies [76]. On the other hand, up to 25% of patients retained severe neurological symptoms despite normalization of hematological deficiency markers [as reported in earlier studies [77,78]. Duration and severity of pre-treatment deficiency symptoms were shown to strongly impact the time-course and degree of recovery after starting the B12 therapy [73,79].
Reversibility of clinical symptoms upon treatment	The recovery of hematological and neurological endpoints to vitamin B12 treatment can vary between patients [68,80], but well-designed studies with adequate follow up time are not available. Among patients with SCD, B12 treatment improved some signs and symptoms in all patients. However some signs and symptoms persisted such as Romberg's sign and mild sensory disturbances in toes and fingers [81,82]. The clinical effect of B12 treatment was better when larger doses were administered more frequently [83]. After B12 treatment, most patients show varying degree of improvement in paresthesia and imbalance of gait [35], complete recovery of stomatitis, sensory neuronopathy improved after 7 weeks (range: 2–32 weeks) [2], while signs of neuropathy and pyramidal improved over few months [35]. In approximately 20% of patients with neurological signs and symptoms, recovery may be only partial or may last more than 3 months after starting the therapy [36]. It can take up to one year for sensory symptoms to resolve after start of B12 therapy [35]. Neuropsychological tests were corrected after 6 weeks of multiple i.m. injections with 1000 µg B12 [84], but micro-structural recovery was delayed compared to functional recovery after 6 weeks of therapy.

Table S7. Topics related to diagnosis and treatment of vitamin B12 deficiency that did not achieve consensus of the panelists according to the study criteria [1].

- Sensory deficits, paresthesia and/or instability of stand and gait could be a mutual manifestation of polyneuropathy and subacute combined degeneration of the spinal cord among patients with B12 deficiency. These two conditions should be differentiated. Patients may be referred for special neurological, neurophysiological, or radiological investigations.
- What to do if the clinical symptoms of a person do not improve after 4-8 weeks of replacement therapy with B12 (either oral or parenteral)?
- Most people with B12 deficiency will need replacement therapy with B12 for many years. What is the view on monitoring the treatment (clinical signs or measuring plasma B12) in people on high-dose oral B12 replacement?
- How to decide on the dose and route of B12 therapy in B12 deficiency?
- The need to recommend prophylactic B12 supplementation to the following risk groups of people (not B12 deficient at present) even without measuring plasma B12. Example of the risk groups are people after various bariatric surgeries, people with diabetes using metformin, people using long-term PPI or acid blockers, people with Parkinson's disease on L-dopa, people with mental illnesses (not caused by B12 deficiency), and people with renal dysfunction.
- Regarding the usefulness of plasma vitamin B12 test, metabolic B12 markers and holotranscobalamin in special circumstances.

[1] Refer to the main manuscript for the authors discussion on aspects that did not achieve consensus among the panelists.

Reference List

[1] Bhatia P, Kulkarni JD, Pai SA. Vitamin B12 deficiency in India: mean corpuscular volume is an unreliable screening parameter. *Natl Med J India* 2012; **25**: 336-8.

[2] Franques J, Chiche L, De Paula AM et al. Characteristics of patients with vitamin B12-responsive neuropathy: a case series with systematic repeated electrophysiological assessment. *Neurol Res* 2019; **41**: 569-76.

[3] Bi Z, Cao J, Shang K, Su Z, Xu S, Liu C. Correlation between anemia and clinical severity in subacute combined degeneration patients. *J Clin Neurosci* 2020; **80**: 11-5.

[4] Cao J, Xu S, Liu C. Is serum vitamin B12 decrease a necessity for the diagnosis of subacute combined degeneration?: A meta-analysis. *Medicine (Baltimore)* 2020; **99**: e19700.

[5] Cao J, Su ZY, Xu SB, Liu CC. Subacute Combined Degeneration: A Retrospective Study of 68 Cases with Short-Term Follow-Up. *Eur Neurol* 2018; **79**: 247-55.

[6] Leishear K, Boudreau RM, Studenski SA et al. Relationship between vitamin B12 and sensory and motor peripheral nerve function in older adults. *J Am Geriatr Soc* 2012; **60**: 1057-63.

[7] Solomon LR. Vitamin B12-responsive neuropathies: A case series. *Nutr Neurosci* 2016; **19**: 162-8.

[8] Warendorf JK, van Doormaal PTC, Vrancken AFJE et al. Clinical relevance of testing for metabolic vitamin B12 deficiency in patients with polyneuropathy. *Nutr Neurosci* 2022; **25**: 2536-46.

[9] Li J, Ren M, Dong A et al. A retrospective study of 23 cases with subacute combined degeneration. *Int J Neurosci* 2016; **126**: 872-7.

[10] Linazi G, Abudureyimu S, Zhang J et al. Clinical features of different stage subacute combined degeneration of the spinal cord. *Medicine (Baltimore)* 2022; **101**: e30420.

[11] Maghsoudlou P, Varlamova J, Pandit J. Patients with unexplained neurological symptoms and signs should be screened for vitamin B12 deficiency regardless of haemoglobin levels. *Eye (Lond)* 2022; **36**: 1124-5.

[12] Gupta K, Jain A, Rohatgi A. An observational study of vitamin b12 levels and peripheral neuropathy profile in patients of diabetes mellitus on metformin therapy. *Diabetes Metab Syndr* 2018; **12**: 51-8.

[13] Kancherla V, Elliott JL, Jr., Patel BB et al. Long-term Metformin Therapy and Monitoring for Vitamin B12 Deficiency Among Older Veterans. *J Am Geriatr Soc* 2017; **65**: 1061-6.

[14] Kos E, Liszek MJ, Emanuele MA, Durazo-Arvizu R, Camacho P. Effect of metformin therapy on vitamin D and vitamin B(1)(2) levels in patients with type 2 diabetes mellitus. *Endocr Pract* 2012; **18**: 179-84.

[15] Jayashri R, Venkatesan U, Rohan M et al. Prevalence of vitamin B(12) deficiency in South Indians with different grades of glucose tolerance. *Acta Diabetol* 2018; **55**: 1283-93.

[16] Bherwani S, Ahirwar AK, Saumya AS et al. The study of association of Vitamin B(12) deficiency in type 2 diabetes mellitus with and without diabetic nephropathy in North Indian Population. *Diabetes Metab Syndr* 2017; **11 Suppl 1**: S365-S368.

[17] Kang D, Yun JS, Ko SH et al. Higher prevalence of metformin-induced vitamin B12 deficiency in sulfonylurea combination compared with insulin combination in patients with type 2 diabetes: a cross-sectional study. *PLoS ONE* 2014; **9**: e109878.

[18] Singh AK, Kumar A, Karmakar D, Jha RK. Association of B12 deficiency and clinical neuropathy with metformin use in type 2 diabetes patients. *J Postgrad Med* 2013; **59**: 253-7.

[19] Chapman LE, Darling AL, Brown JE. Association between metformin and vitamin B(12) deficiency in patients with type 2 diabetes: A systematic review and meta-analysis. *Diabetes Metab* 2016; **42**: 316-27.

[20] Haeusler S, Parry-Strong A, Krebs JD. The prevalence of low vitamin B12 status in people with type 2 diabetes receiving metformin therapy in New Zealand--a clinical audit. *N Z Med J* 2014; **127**: 8-16.

[21] Ting RZ, Szeto CC, Chan MH, Ma KK, Chow KM. Risk factors of vitamin B(12) deficiency in patients receiving metformin. *Arch Intern Med* 2006; **166**: 1975-9.

[22] de Jager J, Kooy A, Lehert P et al. Long term treatment with metformin in patients with type 2 diabetes and risk of vitamin B-12 deficiency: randomised placebo controlled trial. *BMJ* 2010; **340**: c2181.

[23] Aroda VR, Edelstein SL, Goldberg RB et al. Long-term Metformin Use and Vitamin B12 Deficiency in the Diabetes Prevention Program Outcomes Study. *J Clin Endocrinol Metab* 2016; **101**: 1754-61.

[24] Miyan Z, Waris N. Association of vitamin B(12) deficiency in people with type 2 diabetes on metformin and without metformin: a multicenter study, Karachi, Pakistan. *BMJ Open Diabetes Res Care* 2020; **8**.

[25] Parry-Strong A, Langdana F, Haeusler S, Weatherall M, Krebs J. Sublingual vitamin B12 compared to intramuscular injection in patients with type 2 diabetes treated with metformin: a randomised trial. *N Z Med J* 2016; **129**: 67-75.

[26] Mirkazemi C, Peterson GM, Tenni PC, Jackson SL. Vitamin B12 deficiency in Australian residential aged care facilities. *J Nutr Health Aging* 2012; **16**: 277-80.

[27] Soh Y, Won CW. Association between frailty and vitamin B12 in the older Korean population. *Medicine (Baltimore)* 2020; **99**: e22327.

[28] Couderc AL, Camalet J, Schneider S et al. Cobalamin deficiency in the elderly: aetiology and management: a study of 125 patients in a geriatric hospital. *J Nutr Health Aging* 2015; **19**: 234-9.

[29] Andres E, Affenberger S, Vinzio S et al. Food-cobalamin malabsorption in elderly patients: clinical manifestations and treatment. *Am J Med* 2005; **118**: 1154-9.

[30] Abrahamsen JF, Monsen AB, Ranhoff AH et al. No association between subnormal serum vitamin B12 and anemia in older nursing home patients. *Eur Geriatr Med* 2020; **11**: 247-54.

[31] den Elzen WP, Westendorp RG, Frolich M, de RW, Assendelft WJ, Gussekloo J. Vitamin B12 and folate and the risk of anemia in old age: the Leiden 85-Plus Study. *Arch Intern Med* 2008; **168**: 2238-44.

[32] den Elzen WP, van der Weele GM, Gussekloo J, Westendorp RG, Assendelft WJ. Subnormal vitamin B12 concentrations and anaemia in older people: a systematic review. *BMC Geriatr* 2010; **10**: 42.

[33] Ates BE, Soysal P, Aydin AE, Dokuzlar O, Kocyigit SE, Isik AT. Vitamin B12 deficiency might be related to sarcopenia in older adults. *Exp Gerontol* 2017; **95**: 136-40.

[34] Willis CD, Elshaug AG, Milverton JL, Watt AM, Metz MP, Hiller JE. Diagnostic performance of serum cobalamin tests: a systematic review and meta-analysis. *Pathology* 2011; **43**: 472-81.

[35] Divate PG, Patanwala R. Neurological manifestations of B(12) deficiency with emphasis on its aetiology. *J Assoc Physicians India* 2014; **62**: 400-5.

[36] Misra UK, Kalita J. Comparison of clinical and electrodiagnostic features in B12 deficiency neurological syndromes with and without antiparietal cell antibodies. *Postgrad Med J* 2007; **83**: 124-7.

[37] Junca J, de Soria PL, Granada ML, Flores A, Marquez E. Detection of early abnormalities in gastric function in first-degree relatives of patients with pernicious anemia. *Eur J Haematol* 2006; **77**: 518-22.

[38] Salinas M, Flores E, Lopez-Garrigos M, Leiva-Salinas C. High frequency of anti-parietal cell antibody (APCA) and intrinsic factor blocking antibody (IFBA) in individuals with severe vitamin B12 deficiency - an observational study in primary care patients. *Clin Chem Lab Med* 2020; **58**: 424-9.

[39] Soykan I, Yakut M, Keskin O, Bektas M. Clinical profiles, endoscopic and laboratory features and associated factors in patients with autoimmune gastritis. *Digestion* 2012; **86**: 20-6.

[40] Hughes JW, Muegge BD, Tobin GS et al. High-risk gastric pathology and prevalent autoimmune diseases in patients with pernicious anemia. *Endocr Pract* 2017; **23**: 1297-303.

[41] Yang GT, Zhao HY, Kong Y, Sun NN, Dong AQ. Correlation between serum vitamin B12 level and peripheral neuropathy in atrophic gastritis. *World J Gastroenterol* 2018; **24**: 1343-52.

[42] Miceli E, Lenti MV, Padula D et al. Common features of patients with autoimmune atrophic gastritis. *Clin Gastroenterol Hepatol* 2012; **10**: 812-4.

[43] Ao M, Tsuji H, Shide K et al. High prevalence of vitamin B-12 insufficiency in patients with Crohn's disease. *Asia Pac J Clin Nutr* 2017; **26**: 1076-81.

[44] Madanchi M, Fagagnini S, Fournier N et al. The Relevance of Vitamin and Iron Deficiency in Patients with Inflammatory Bowel Diseases in Patients of the Swiss IBD Cohort. *Inflamm Bowel Dis* 2018; **24**: 1768-79.

[45] Yakut M, Ustun Y, Kabacam G, Soykan I. Serum vitamin B12 and folate status in patients with inflammatory bowel diseases. *Eur J Intern Med* 2010; **21**: 320-3.

[46] Schosler L, Christensen LA, Hvas CL. Symptoms and findings in adult-onset celiac disease in a historical Danish patient cohort. *Scand J Gastroenterol* 2016; **51**: 288-94.

[47] Nguyen T, van Oijen MG, Janssen MJ, Laheij RJ, Jansen JB, van AH. Vitamin B12 deficiency in patients with upper gastrointestinal symptoms in the Mekong Delta, Vietnam. *Dig Liver Dis* 2006; **38**: 438-9.

[48] Rozgony NR, Fang C, Kuczmarski MF, Bob H. Vitamin B(12) deficiency is linked with long-term use of proton pump inhibitors in institutionalized older adults: could a cyanocobalamin nasal spray be beneficial? *J Nutr Elder* 2010; **29**: 87-99.

[49] Schiavon CA, Bhatt DL, Ikeoka D et al. Three-Year Outcomes of Bariatric Surgery in Patients With Obesity and Hypertension : A Randomized Clinical Trial. *Ann Intern Med* 2020; **173**: 685-93.

[50] Schijns W, Schuurman LT, Melse-Boonstra A, van Laarhoven CJHM, Berends FJ, Aarts EO. Do specialized bariatric multivitamins lower deficiencies after RYGB? *Surg Obes Relat Dis* 2018; **14**: 1005-12.

[51] Vilarrasa N, Fabregat A, Toro S et al. Nutritional deficiencies and bone metabolism after endobarrier in obese type 2 patients with diabetes. *Eur J Clin Nutr* 2018; **72**: 1447-50.

[52] Bilici A, Sonkaya A, Ercan S et al. The changing of serum vitamin B12 and homocysteine levels after gastrectomy in patients with gastric cancer: do they associate with clinicopathological factors? *Tumour Biol* 2015; **36**: 823-8.

[53] Hu Y, Kim HI, Hyung WJ et al. Vitamin B(12) deficiency after gastrectomy for gastric cancer: an analysis of clinical patterns and risk factors. *Ann Surg* 2013; **258**: 970-5.

[54] Aoyama T, Hara K, Maezawa Y et al. Clinical Course of Vitamin B12 Deficiency and Associated Risk Factors in Patients After Total Gastrectomy for Gastric Cancer. *Anticancer Res* 2023; **43**: 689-94.

[55] Vargas-Ruiz AG, Hernandez-Rivera G, Herrera MF. Prevalence of iron, folate, and vitamin B12 deficiency anemia after laparoscopic Roux-en-Y gastric bypass. *Obes Surg* 2008; **18**: 288-93.

[56] Antoine D, Li Z, Quilliot D et al. Medium term post-bariatric surgery deficit of vitamin B12 is predicted by deficit at time of surgery. *Clin Nutr* 2021; **40**: 87-93.

[57] Adali Y, Binnetoglu K. Evaluation of the response to vitamin B12 supplementation in patients with atrophy in sleeve gastrectomy materials. *Cir Cir* 2022; **90**: 17-23.

[58] Aoyama T, Maezawa Y, Cho H et al. Phase II Study of a Multi-center Randomized Controlled Trial to Evaluate Oral Vitamin B12 Treatment for Vitamin B12 Deficiency After Total Gastrectomy in Gastric Cancer Patients. *Anticancer Res* 2022; **42**: 3963-70.

[59] Mahawar KK, Reid A, Graham Y et al. Oral Vitamin B(12) Supplementation After Roux-en-Y Gastric Bypass: a Systematic Review. *Obes Surg* 2018; **28**: 1916-23.

[60] Smelt HJ, Pouwels S, Smulders JF. Different Supplementation Regimes to Treat Perioperative Vitamin B12 Deficiencies in Bariatric Surgery: a Systematic Review. *Obes Surg* 2017; **27**: 254-62.

[61] Majumder S, Soriano J, Louie CA, Dasanu CA. Vitamin B12 deficiency in patients undergoing bariatric surgery: preventive strategies and key recommendations. *Surg Obes Relat Dis* 2013; **9**: 1013-9.

[62] Engebretsen KV, Blom-Hogestol IK, Hewitt S et al. Anemia following Roux-en-Y gastric bypass for morbid obesity; a 5-year follow-up study. *Scand J Gastroenterol* 2018; **53**: 917-22.

[63] Ramos RJ, Mottin CC, Alves LB, Mulazzani CM, Padoin AV. Vitamin B12 supplementation orally and intramuscularly in people with obesity undergoing gastric bypass. *Obes Res Clin Pract* 2021; **15**: 177-9.

[64] Smelt HJ, Pouwels S, Said M, Berghuis KA, Boer AK, Smulders JF. Comparison Between Different Intramuscular Vitamin B(12) Supplementation Regimes: a Retrospective Matched Cohort Study. *Obes Surg* 2016; **26**: 2873-9.

[65] Moore CE, Sherman V. Effectiveness of B vitamin supplementation following bariatric surgery: rapid increases of serum vitamin B12. *Obes Surg* 2015; **25**: 694-9.

[66] Namikawa T, Maeda M, Yokota K et al. Enteral Vitamin B12 Supplementation Is Effective for Improving Anemia in Patients Who Underwent Total Gastrectomy. *Oncology* 2021; **99**: 225-33.

[67] Sanz-Cuesta T, Escortell-Mayor E, Cura-Gonzalez I et al. Oral versus intramuscular administration of vitamin B12 for vitamin B12 deficiency in primary care: a pragmatic, randomised, non-inferiority clinical trial (OB12). *BMJ Open* 2020; **10**: e033687.

[68] Bolaman Z, Kadikoylu G, Yukselen V, Yavasoglu I, Barutca S, Senturk T. Oral versus intramuscular cobalamin treatment in megaloblastic anemia: a single-center, prospective, randomized, open-label study. *Clin Ther* 2003; **25**: 3124-34.

[69] Kalita J, Agarwal R, Chandra S, Misra UK. A study of neurobehavioral, clinical psychometric, and P3 changes in vitamin B12 deficiency neurological syndrome. *Nutr Neurosci* 2013; **16**: 39-46.

[70] Tu MC, Lo CP, Chen CY, Huang CF. Correlation of Tc-99 m ethyl cysteinate dimer single-photon emission computed tomography and clinical presentations in patients with low cobalamin status. *BMC Neurol* 2015; **15**: 251.

[71] Gupta PK, Garg RK, Gupta RK et al. Diffusion tensor tractography and neuropsychological assessment in patients with vitamin B12 deficiency. *Neuroradiology* 2014; **56**: 97-106.

[72] Reynolds E. Vitamin B12, folic acid, and the nervous system. *Lancet Neurol* 2006; **5**: 949-60.

[73] Puri V, Chaudhry N, Goel S, Gulati P, Nehru R, Chowdhury D. Vitamin B12 deficiency: a clinical and electrophysiological profile. *Electromyogr Clin Neurophysiol* 2005; **45**: 273-84.

[74] Aaron S, Kumar S, Vijayan J, Jacob J, Alexander M, Gnanamuthu C. Clinical and laboratory features and response to treatment in patients presenting with vitamin B12 deficiency-related neurological syndromes. *Neurol India* 2005; **53**: 55-8.

[75] Saperstein DS, Wolfe GI, Gronseth GS et al. Challenges in the identification of cobalamin-deficiency polyneuropathy. *Arch Neurol* 2003; **60**: 1296-301.

[76] Misra UK, Kalita J, Das A. Vitamin B12 deficiency neurological syndromes: a clinical, MRI and electrodiagnostic study. *Electromyogr Clin Neurophysiol* 2003; **43**: 57-64.

[77] Stabler SP, Allen RH, Savage DG, Lindenbaum J. Clinical spectrum and diagnosis of cobalamin deficiency. *Blood* 1990; **76**: 871-81.

[78] Lindenbaum J, Healton EB, Savage DG et al. Neuropsychiatric disorders caused by cobalamin deficiency in the absence of anemia or macrocytosis. *N Engl J Med* 1988; **318**: 1720-8.

[79] Healton EB, Savage DG, Brust JC, Garrett TJ, Lindenbaum J. Neurologic aspects of cobalamin deficiency. *Medicine (Baltimore)* 1991; **70**: 229-45.

[80] Butler CC, Vidal-Alaball J, Cannings-John R et al. Oral vitamin B12 versus intramuscular vitamin B12 for vitamin B12 deficiency: a systematic review of randomized controlled trials. *Fam Pract* 2006; **23**: 279-85.

[81] Jain KK, Malhotra HS, Garg RK, Gupta PK, Roy B, Gupta RK. Prevalence of MR imaging abnormalities in vitamin B12 deficiency patients presenting with clinical features of subacute combined degeneration of the spinal cord. *J Neurol Sci* 2014; **342**: 162-6.

[82] Xiao CP, Ren CP, Cheng JL et al. Conventional MRI for diagnosis of subacute combined degeneration (SCD) of the spinal cord due to vitamin B-12 deficiency. *Asia Pac J Clin Nutr* 2016; **25**: 34-8.

[83] Jhunjhnuwala D, Tanglay O, Briggs NE, Yuen MTY, Huynh W. Prognostic indicators of subacute combined degeneration from B12 deficiency: A systematic review. *PM R* 2022; **14**: 504-14.

[84] Roy B, Trivedi R, Garg RK, Gupta PK, Tyagi R, Gupta RK. Assessment of functional and structural damage in brain parenchyma in patients with vitamin B12 deficiency: A longitudinal perfusion and diffusion tensor imaging study. *Magn Reson Imaging* 2015; **33**: 537-43.
