# Supplementary figures and images for: Diagnosis, Treatment and Long-Term Management of Vitamin B12 Deficiency in Adults: A Delphi Expert Consensus
Source: J Clin Med. 2024 Apr 10;13(8):2176. doi: 10.3390/jcm13082176 (PMC11050313; doi:10.3390/jcm13082176)

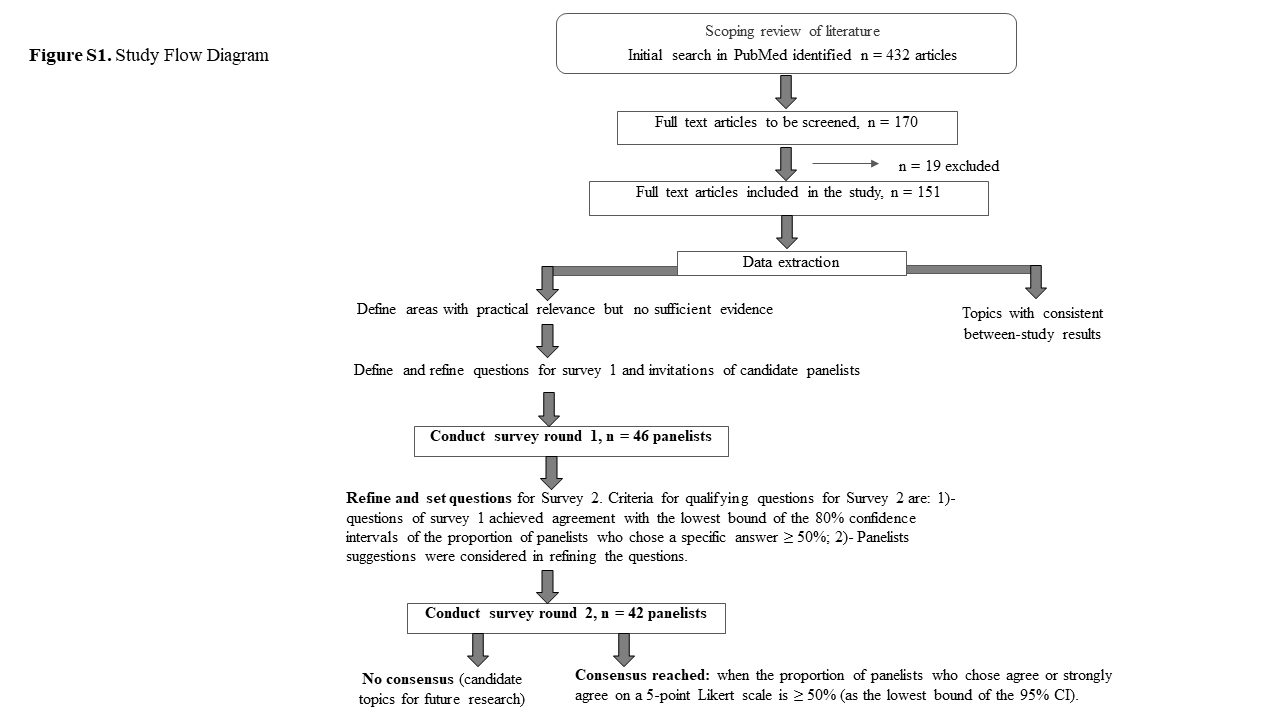

Supplement: Supplementary file 1 [file jcm-13-02176-s001.zip › Figure S1.TIF]

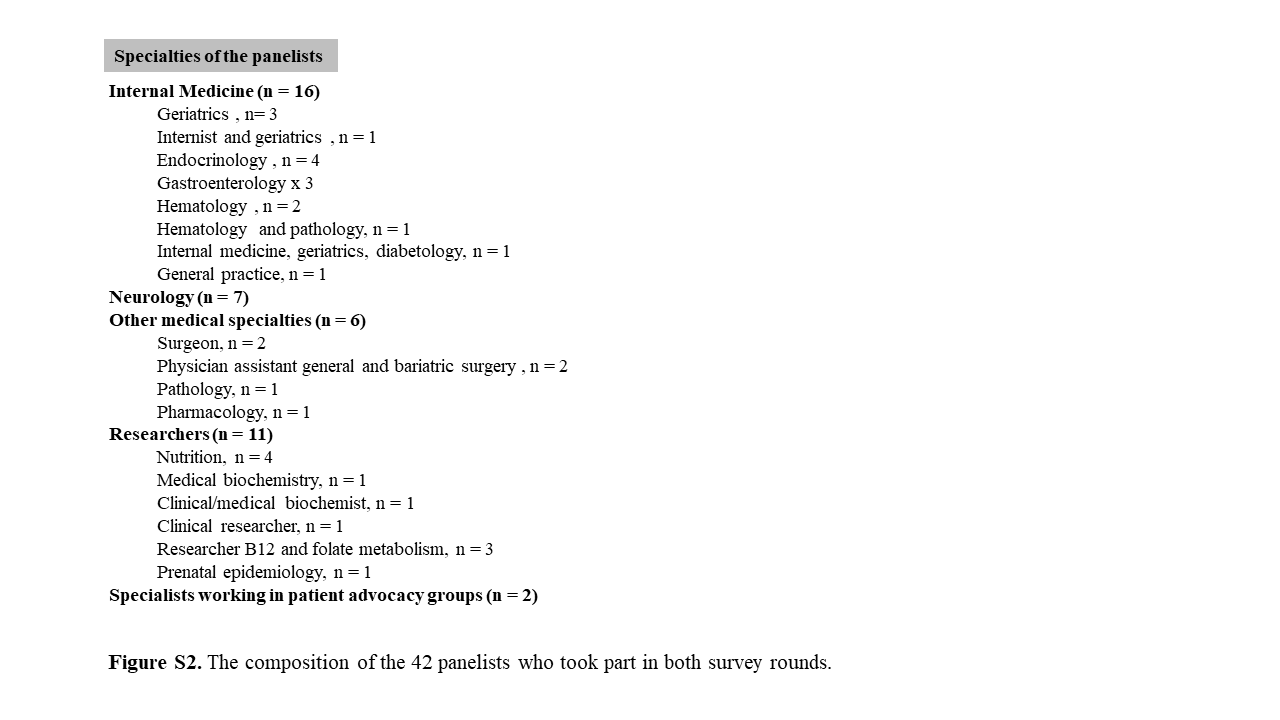

Supplement: Supplementary file 1 [file jcm-13-02176-s001.zip › Figure S2.TIF]

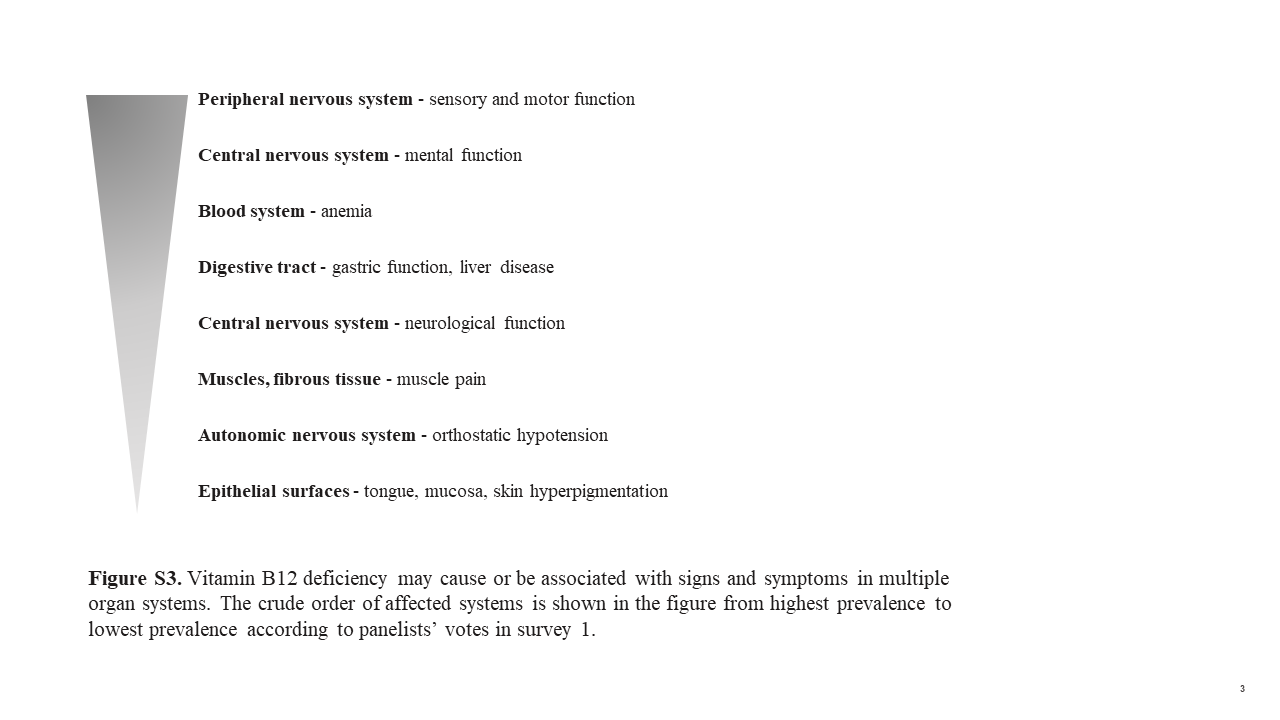

Supplement: Supplementary file 1 [file jcm-13-02176-s001.zip › Figure S3.TIF]
